# Intra-Articular Corticosteroids in Addition to Exercise for Reducing Pain Sensitivity in Knee Osteoarthritis: Exploratory Outcome from a Randomized Controlled Trial

**DOI:** 10.1371/journal.pone.0149168

**Published:** 2016-02-12

**Authors:** Alberto Soriano-Maldonado, Louise Klokker, Cecilie Bartholdy, Elisabeth Bandak, Karen Ellegaard, Henning Bliddal, Marius Henriksen

**Affiliations:** 1 Department of Physical Education and Sport, Faculty of Sport Sciences, University of Granada, Granada, Spain; 2 The Parker Institute, Copenhagen University Hospital Bispebjerg and Frederiksberg, Frederiksberg, Denmark; 3 Department of Physical and Occupational Therapy, Copenhagen University Hospital Bispebjerg and Frederiksberg, Frederiksberg, Denmark; VU University Medical Center, NETHERLANDS

## Abstract

**Objective:**

To assess the effects of one intra-articular corticosteroid injection two weeks prior to an exercise-based intervention program for reducing pain sensitivity in patients with knee osteoarthritis (OA).

**Design:**

Randomized, masked, parallel, placebo-controlled trial involving 100 participants with clinical and radiographic knee OA that were randomized to one intra-articular injection on the knee with either 1 ml of 40 mg/ml methylprednisolone (corticosteroid) dissolved in 4 ml lidocaine (10 mg/ml) or 1 ml isotonic saline (placebo) mixed with 4 ml lidocaine (10 mg/ml). Two weeks after the injections all participants undertook a 12-week supervised exercise program. Main outcomes were changes from baseline in pressure-pain sensitivity (pressure-pain threshold [PPT] and temporal summation [TS]) assessed using cuff pressure algometry on the calf. These were exploratory outcomes from a randomized controlled trial.

**Results:**

A total of 100 patients were randomized to receive either corticosteroid (n = 50) or placebo (n = 50); 45 and 44, respectively, completed the trial. Four participants had missing values for PPT and one for TS at baseline; thus modified intention-to-treat populations were analyzed. The mean group difference in changes from baseline at week 14 was 0.6 kPa (95% CI: -1.7 to 2.8; P = 0.626) for PPT and 384 mm×sec (95% CI: -2980 to 3750; P = 0.821) for TS.

**Conclusions:**

These results suggest that adding intra-articular corticosteroid injection 2 weeks prior to an exercise program does not provide additional benefits compared to placebo in reducing pain sensitivity in patients with knee OA.

**Trial Registration:**

EU clinical trials (EudraCT): 2012-002607-18

## Introduction

Osteoarthritis (OA) of the knee involves inflammation and structural changes of the joint, and is one of the main causes of pain and disability, leading to massive health care costs [1–2]. It has been shown that joint damage does not strongly correlate with pain severity in patients with knee OA [3], and previous studies, including a meta-analysis of 41 studies, reinforced the idea that central sensitization is a mechanism that may at least partially explain pain and symptoms severity in this population [4–5].

Recent guidelines on the management of knee OA highlight the need of research on the combination of non-pharmacological and pharmacological treatment modalities [6]. Previous research from our group revealed that supervised exercise therapy focused on strength and coordination exercises reduced pressure-pain sensitivity in patients with knee OA [7]. It has also been suggested that pain sensitivity improves (for at least 2 weeks) following an intra-articular steroid injection [8]. Therefore, we hypothesized that an intra-articular corticosteroid injection preceding an exercise intervention program may provide a window of opportunity for a greater exercise-related adaptations and consequent improvement on pain sensitivity.

This study is an exploratory sub-study of a randomized double-blinded, placebo-controlled clinical trial [9] aiming at investigating the clinical effects of intra-articular steroid injection therapy given 2 weeks prior to a 12-week exercise program in individuals with knee OA. The clinical results suggested comparable effects of steroid and placebo given prior to exercise on knee OA symptoms [9], yet it remains unknown if the combination yields beneficial effects on measures of pain sensitivity.

The aim of this exploratory sub-study was to assess the potential of intra-articular corticosteroids injected two weeks prior to an exercise intervention program to provide additional benefits (over placebo) on pain sensitivity in patients with knee OA.

## Materials and Methods

The detailed procedures and results from the primary outcome of the trial are reported elsewhere [9]. The detailed original study protocol in Danish is included as supporting information (S1 Protocol) and a retrospective translation of the main sections is included as supporting information (S2 Protocol). In brief, we undertook a participant, care provider, and outcome assessor blinded, two-arm, parallel-group, randomized, and placebo-controlled trial, running over 26 weeks from October 1, 2012, to April 2, 2014. The study protocol was registered on August 7, 2012, before enrolment of participants started (EudraCT number: 2012-002607-18) and no deviations from the protocol occurred. The Danish Health and Medicines Authority and the Regional Health Research Ethics Committee reviewed and approved the protocol on August 30, 2012. All participants gave their oral and written informed consent to participate in the trial. This article follows the CONSORT reporting guidelines [10]; see CONSORT checklist included as supporting information (S1 Checklist). The authors confirm that all ongoing and related trials for this drug/intervention are registered. The funding source had no role in any stage of the study.

### Setting and eligibility criteria

Participants were recruited from the OA outpatient clinic from the Copenhagen University Hospitals at Bispebjerg and Frederiksberg, Copenhagen, Denmark. A comprehensive description of the recruitment process and inclusion/exclusion criteria is published elsewhere [9]. Briefly, inclusion criteria comprised being 40 years or older and having a radiographic confirmation of a clinical diagnosis of tibiofemoral OA, clinical signs of localized knee inflammation, knee pain during walking (score of >4 on a scale of 0–10 points), and a body mass index ≤35. Potential participants who had corticosteroid injections or participated in exercise therapy within the previous 3 months, were excluded, as were those who used oral corticosteroids within the previous 4 weeks or had inflammatory arthritis, a history of knee arthroplasty, conditions precluding exercise participation, contraindications to corticosteroids, regional pain syndromes (e.g., fibromyalgia), or spinal nerve root compression syndromes.

### Procedures

A telephone screening was conducted, and potentially eligible participants were invited to a clinical screening examination performed by a rheumatologist (HB). During the examination, eligibility criteria were assessed including a standardized, semiflexed, weight-bearing posterior-anterior knee radiograph to confirm the diagnosis of knee OA. At inclusion, each participant chose the most symptomatic knee as target knee for all evaluations, and baseline assessments were undertaken. Subsequently, participants were randomized and an independent blinded researcher performed the injections. To allow expected maximal effect of the corticosteroid [11], the exercise program began 2 weeks after the injection and continued for 12 weeks [9]. Both groups (corticosteroid and placebo) participated in the same exercise classes.

### Interventions

The injections were performed with a 25 gauge (38 mm) needle and a 10 ml Luer-lock syringe under real time ultrasonographic guidance (Logic E9, General Electrics Medical System with a 15 MHz linear array transducer, Milwaukee, WI, USA) by an experienced specialist in musculoskeletal sonography to ensure correct bolus deposition in the joint cavity. Participants in the corticosteroid group received an intra-articular injection with 1 ml methylprednisolone (40 mg Depo-Medrol^®^, Pfizer) dissolved in 4 ml lidocaine (10 mg/ml, SAD) and participants in the placebo group received an injection with 1 ml isotonic saline mixed with 4 ml lidocaine (10 mg/ml, SAD).

A comprehensive description of the exercise program, including specific exercises, progressions, and additional details are described elsewhere [7]. The program consisted of group-based supervised exercise sessions (3 times/week during 12 weeks) in which participants joined the group consecutively as they were enrolled. The sessions lasted 1 hour (including a warm-up and a cool-down phase) and consisted of circuit training focused on strength and coordination exercises of the trunk, hips and knees [7]. The exercise program was identical to that previously shown to reduce pain sensitivity in knee OA [7].

### Outcome measures

Outcomes were measured at baseline (before randomization and injection), at week 14 (i.e. immediately after the 12-week exercise program; primary trial endpoint), and at week 26 (i.e. after 12 weeks of no attention). The primary outcome measure of the parent trial [9] was the pain subscale of the Knee Injury and Osteoarthritis Outcome Score (KOOS) questionnaire [12]. In the present study, we present measures of pressure pain sensitivity (pressure pain threshold [PPT] and temporal summation [TS]), which are exploratory outcomes of the parent trial (i.e. the study was not specifically designed to evaluate pain sensitivity) and main outcomes of this study.

Pressure-pain sensitivity was estimated by computerized cuff pressure algometry [13], which consists of a double-chamber tourniquet cuff (VBM Medizin-technik GmbH) and a computer-controlled air compressor (DoloCuff, Unique Electronic Aps). The cuff was wrapped around the calf at the bulky part of the gastrocnemius muscle on the leg of the nominated target knee. Both chambers of the cuff were automatically and simultaneously inflated at a compression rate of 1 kPa/second until the patient reported that the sensation of pressure started to become painful. The pressure at this point defined the PPT (measured in kPa). The PPT was registered 3 times separated by at least 60 seconds, and the average was used for the analyses. The intraclass correlation coefficient for the PPT using cuff algometry is 0.72 (95% confidence interval [CI] 0.64 to 0.87) with a minimal detectable change of 2.2 kPa, which is similar to other PPT assessment methods in knee OA [14]. Temporal summation of pressure–pain was assessed on the same site as the PPT using constant pressure stimulation at 125% of the PPT during 6 minutes. Participants continuously rated their pain intensity during the test on an electronic 0–100-mm visual analog scale (VAS), with 0 and 100 mm anchored as “no pain” and “worst pain imaginable”, respectively. The VAS signal was sampled by a computer at 10-Hz. To quantify TS of pressure–pain, the area under the time-VAS curve was calculated and expressed in mm×seconds. To account for changes in PPT at follow-up potentially blurring effects on TS, the same constant pressure used at baseline was applied at follow-up. Reliability coefficients and minimal detectable change in TS are unknown.

### Sample size

The sample size (n = 100) was calculated for the primary outcome of the hosting trial under the assumption that the corticosteroid group would improve at least 10 units more on the primary study outcome (changes from baseline in the KOOS pain subdomain) than the placebo group at the primary end-point (week 14), using a 2-tailed test, with a SD of 15 points, 91% power and alpha error of 5% [9].

### Randomization, treatment allocation and blinding

After baseline assessments, participants were randomized in permuted blocks of 2 to 6 to receive either an intra-articular corticosteroid or placebo injection. A blinded study member created a computer generated randomization sequence, before patients were enrolled, to allocate participants in permuted blocks of 2 to 6 to either of the groups (1:1). Individual allocations were held in sealed, opaque, and consecutively numbered envelopes.

Solely an un-blinded study nurse prepared the syringes in the absence of participants and blinded study staff. Since the corticosteroid liquid is milky white and the saline is clear, the syringes were masked by opaque tape to prevent disclosure of the content during the injection procedure.

### Statistical analysis

Main analyses were performed on the modified intention-to-treat (ITT) population, including all randomized participants (retained in the group they were allocated to). Missing data were replaced using multiple imputations with age, gender, BMI, baseline values, and group allocations (masked) as predictors. A blinded investigator (ASM) undertook the initial data handling and all hypothesis testing.

We analyzed the PPT and TS data using repeated measures mixed linear models including participants as a random effect, with fixed factors for group (2 levels; corticosteroid or placebo) and week (2 levels; week 14 and 26) and the corresponding interactions (i.e. the model was directly run with full interaction). The models were controlled for baseline values by including them as covariates. The diagnostic of the linear model(s) was checked through the analysis of the predicted values and the studentized residuals. Results are expressed as estimates of the between group differences in changes from baseline at weeks 14 and 26 with 95% CI. Details of the mixed models (SAS code and output) are provided as supporting information (S1 SAS Output).

To test the robustness of the main analysis, sensitivity analyses were undertaken on the ITT basis using baseline observation carried forward (BOCF) imputation, and the complete-case population (no imputations).

The statistical analysis was performed with SAS statistical software (version 9.3), and statistical significance was set at 5%. The data set is available (S1 Data).

## Results

**Fig 1:**
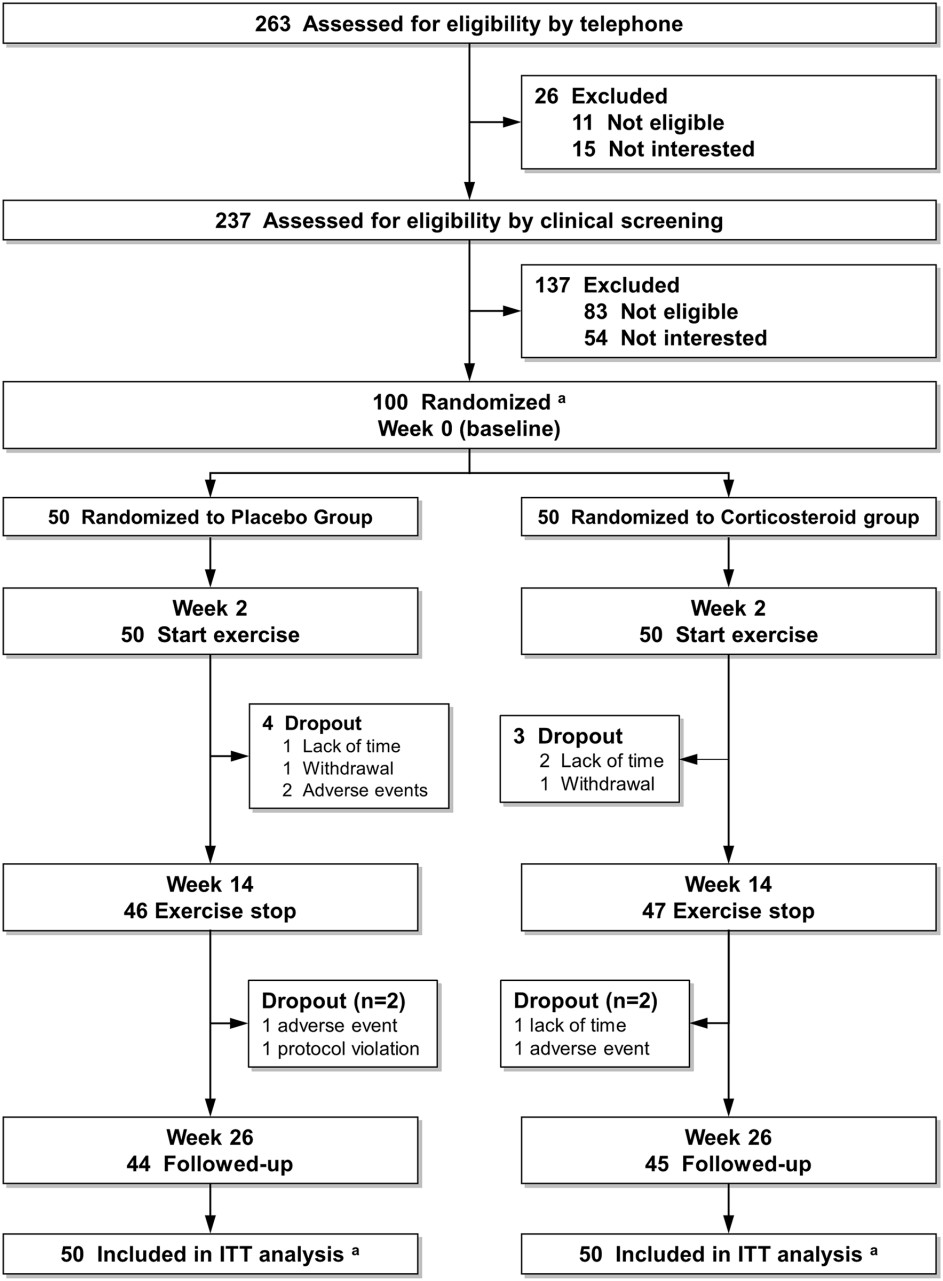
CONSORT diagram showing patient flow through the Trial. ^a^ Indicates intention-to-treat (ITT) population. Modified ITT-1 population was used for pressure-pain threshold analyses, including the 99 participants with valid baseline values [49 in the placebo group and 50 in the corticosteroid group]. Modified ITT-2 was used for temporal summation analyses, including the 96 participants with valid baseline values [48 in the placebo group and 48 in the corticosteroid group].

A total of 100 participants were randomized (50 to each group), received allocated injection and defined the ITT population. The CONSORT flow chart of study participants and the rationale for undertaken modified ITT populations is presented in Fig 1. The groups were balanced at baseline (see Henriksen et al. [9] and table 1).

**Table 1:** Baseline characteristics of the study participants.

		Intervention arms	
	Total sample (n = 100)	Placebo (n = 50)	Corticosteroid (n = 50)
Female (no., %)	61 (61%)	33 (66%)	28 (56%)
Age, y	63.4 (9.3)	65.5 (8.3)	61.3 (9.9)
Height, m	1.71 (0.10)	1.69 (0.09)	1.73 (0.10)
Weight, kg	84.6 (13.2)	82.8 (11.4)	86.5 (14.8)
BMI, kg/m^2^	28.9 (3.6)	28.9 (3.3)	29.0 (3.9)
Radiographic severity, KL, 0–4 †			
Grade 1 (no., %)	4 (4)	0 (0)	4 (8)
Grade 2 (no., %)	39 (39)	18 (36)	21 (42)
Grade 3 (no., %)	32 (32)	17 (34)	15 (30)
Grade 4 (no., %)	25 (25)	15 (30)	10 (20)
PPT, kPa	18.5 (6.4)	18.7 (7.0) ф	18.3 (5.8)
TS, mm × sec	14,423 (8024)	15,331 (7823)‡	13,516 (8201)‡

Values are mean (standard deviation; SD) unless otherwise indicated; BMI, body mass index; PPT, pressure pain threshold; TS, temporal summation.

^†^ Data are number of participants (%).

^ф^ n = 49 (due to technical problems).

^‡^ n = 48 (due to technical problems).

In the placebo group, 4 participants were lost to follow-up at week 14 and 1 at week 26; further, 1 participant received a corticosteroid injection outside of the study between week 14 and 26 and was therefore excluded from further assessments (protocol violation). In the corticosteroid group, 1 participant withdrew at the beginning of the exercise program, 2 were lost to follow-up at week 14, and 2 were lost to follow-up at week 26. Hence, 89 (44 placebo/45 corticosteroid) participants completed the study. Some data were missing at follow-up visits (week 26; i.e. PPT and TS data from 3 participants in the placebo group, and TS data from 2 participants in the corticosteroid group) due to technical problems.

**Fig 2:**
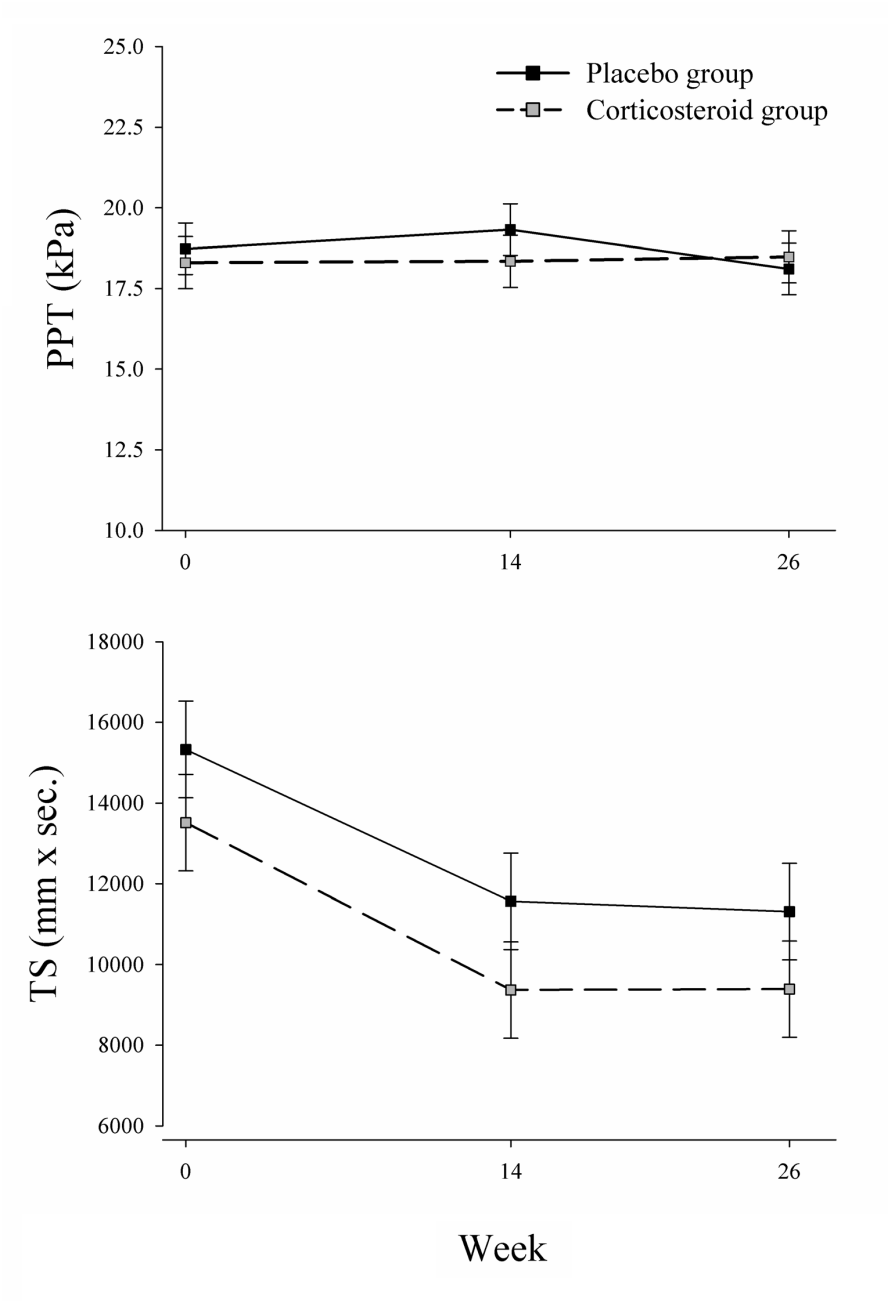
Group differences in pressure-pain threshold (PPT) and temporal summation (TS) at baseline, week 14 (end of the exercise program; primary endpoint) and week 26 (end of the study). PPT, pressure-pain threshold; TS, temporal summation.

The PPT and TS scores in the placebo and corticosteroid groups at baseline, week 14 and week 26 are presented in Fig 2. At baseline, the mean PPT was 18.7 kPa (SD 7.0) in the placebo group and 18.3 kPa (SD 5.8) in the corticosteroid group, and the mean TS was 15,331 mm × sec (SD 7823) in the placebo group and 13,516 mm × sec (SD 8201) in the corticosteroid group (table 2). There were no significant group differences between changes in PPT or TS either at week 14 or week 26 (all, P>0.05; table 2). Sensitivity analyses corroborated these results (table 3). In addition, there was no overall benefit to the pain sensitivity measures regardless of allocation (main effect of week: P = 0.45 and P = 0.89 for PPT and TS, respectively).

**Table 2:** Comparison of pressure-pain threshold (PPT) and temporal summation (TS) changes at week 14 and week 26 after randomization.

Change from baseline	Intervention		Mean difference (95%CI)	P
	Placebo	Corticosteroid		
	mean (SE)	mean (SE)		
	**n = 49**	**n = 50**		
PPT, kPa^‡^ (at week 14)	0.6 (0.8)	0.0 (0.8)	0.6 (-1.7 to 2.8)	0.626
PPT, kPa ^‡^ (at week 26)	-0.6 (0.8)	0.2 (0.8)	-0.8 (-3.0 to 1.4)	0.480
	**n = 48**	**n = 48**		
TS, mm × sec^ǂ^ (at week 14)	-3764 (1196)	-4149 (1196)	384 (-2980 to 3750)	0.821
TS, mm × sec ^ǂ^ (at week 26)	-4020 (1196)	-4128 (1196)	109 (-3256 to 3474)	0.949

SE, standard error; CI, confidence interval.

^‡^ Modified intention to treat 1.

^ǂ^ Modified intention to treat 2.

**Table 3:** Sensitivity analyses: Comparison of pressure-pain threshold (PPT) and temporal summation (TS) changes at weeks 14 and 26 using baseline observation carried forward (BOCF) imputation and complete-case population (no imputations).

Change from baseline in	BOCF				Complete-case			
	Intervention (mean, SE)		Mean difference (95%CI)	P	Intervention (mean, SE)		Mean difference (95%CI)	P
	Placebo	Corticosteroid			Placebo	Corticosteroid		
	**n = 49**	**n = 50**			**n = 49**	**n = 50**		
PPT, kPa (week 14)	1.1 (0.8)	0.2 (0.8)	0.9 (-1.2 to 3.0)	0.410	1.2 (0.8)	0.3 (0.8)	0.9 (-1.4 to 3.2)	0.439
PPT, kPa (week 26)	-0.8 (0.8)	-0.0 (0.8)	-0.7 (-2.8 to 1.4)	0.510	-0.8 (0.9)	-0.1 (0.8)	-0.8 (-3.2 to 1.6)	0.511
	**n = 48**	**n = 48**			**n = 48**	**n = 48**		
TS, mm × sec (week 14)	-2814 (1216)	-3130 (1216)	316 (-3105 to 3738)	0.860	-2993 (1284)	-3443 (1280)	450 (-3168 to 4068)	0.805
TS, mm × sec (week26)	-2738 (1216)	-2687 (1216)	-51 (-3473 to 3370)	0.980	-3637 (1344)	-3123 (1303)	-514 (-4248 to 3219)	0.785

SE, standard error; CI, confidence interval.

## Discussion

The results of the present study do not support the hypothesis that one intra-articular corticosteroid injection 2 weeks prior to an exercise-based intervention program provides additional benefits on pain sensitivity in comparison to placebo in patients with knee OA.

To the best of our knowledge, this is the first study examining the potential synergistic effects of steroid and exercise for reducing pain sensitivity in knee OA. Our results revealed that the steroid injection did not provide any further benefit. These findings are in line with the primary results of this trial showing no group differences either in pain, physical function or inflammation markers [9]. Although it has been suggested that pain sensitivity improves for at least 2 weeks following an intra-articular steroid injection [8], it is not clear what the actual extent of the corticosteroids effects is. It is therefore possible that the exercise program might have begun when the maximal window of opportunity for optimal improvement was exhausted. Future randomized trials using different time points for the beginning of the exercise intervention are warranted.

Previous research from our group showed that PPT and TS significantly improved in patients with knee OA following the same exercise intervention in comparison to a control group (no exercise) [7]. In the present study, however, in this study we did not observe significant intra-group improvements in pain sensitivity measures following the exercise program (despite non-significant improvements [25 to 30%] in TS) in either group. Potential baseline characteristics that might partially explain the different results are a higher proportion of males (39% vs. 20%) and baseline KOOS scores slightly higher (between 2.0 and 7.8 units across subscales) in the current trial [9] compared to the previous one [7]. Also, the proportion of mild and severe radiographic grades was slightly different between the groups (table 1), and it cannot be completely ruled out that radiographic OA severity could affect treatment response on pain sensitivity. However, in experimental studies radiographic knee OA severity is not associated with pain sensitivity [15].

This study had limitations. The dose of corticosteroid (40 mg/mL) used was in the mid-range for knee joints and was chosen because it is the standard procedure at our institution. Further research is warranted to determine whether a higher dose might produce differential results. In addition, different timings for the corticosteroids injection before exercising are warranted. However, the mean group differences in either of the outcomes are so small that both treatments (placebo or steroid) are most likely equivalent. Pain sensitivity was not measured at week 2 (i.e. prior to the beginning of the exercise program) and there might have been undetected between-group differences due to the corticosteroids alone. However, this study aimed at assessing the combined effect of corticosteroid and exercise. Since this study presents exploratory outcomes that may differ according to the above-mentioned parameters, the generalizability of our findings is unknown and future studies are warranted.

## Conclusions

Our results indicate that one intra-articular corticosteroid injection 2 weeks prior to an exercise intervention program does not provide any additional benefit compared to placebo in reducing pain sensitivity in patients with knee OA.

## Supporting Information

S1 Checklist (DOC)

S1 Data (XLSX)

S1 Protocol (PDF)

S2 Protocol (DOC)

S1 SAS Output (TXT)
