# Supplementary material for: Intra-Articular Corticosteroids in Addition to Exercise for Reducing Pain Sensitivity in Knee Osteoarthritis: Exploratory Outcome from a Randomized Controlled Trial
Source: PLoS One. 2016 Feb 12;11(2):e0149168. doi: 10.1371/journal.pone.0149168 (PMC4752217; doi:10.1371/journal.pone.0149168)
Supplement: S1 Protocol — (PDF) [file pone.0149168.s003.pdf]

PROTOKOL

TITEL: KOMBINERET INTRAARTIKULÆR CORTICOSTEROID OG FYSIOTERAPEUTISK TRÆNING HOS PATIENTER MED KNÆARTROSE: ET RANDOMISERET KLINISK FORSØG

ENGELSK TITEL: COMBINED INTRA ARTICULAR CORTICOSTEROID AND PHYSIOTHERAPEUTIC EXERCISE IN PATIENTS WITH OSTEOARTHRITIS OF THE KNEE: A RANDOMISED CLINICAL TRIAL

AKRONYM: PEX-4

PROTOKOLNUMMER: 101.04

EudraCT-nummer: 2012-002607-18

FORSØGSLEDER & SPONSOR: Marius Henriksen[1], fysioterapeut, ph.d.
STUDIEKOORDINATOR: Louise Klokker[1], fysioterapeut, cand. scient. san.
KLINISK ANSVARLIG & INVESTIGATOR: Henning Bliddal[1], professor, overlæge, dr.med.
STATISTIKER: Robin Christensen[1], cand. scient., ph.d.
KLINISK FARMACEUT: Simon Tarp[1], cand. pharm.
MONITOR: Københavns Universitetshospitals GCP-enhed

ØVRIGE VIDENSKABELIGE MEDARBEJDERE:
Bente Danneskiold-Samsøe[1], professor, overlæge, dr.med.
Søren Torp-Pedersen[1], overlæge
Mikael Boesen[1,2], læge

AFFILIERINGER:
[1] Parker Instituttet, Reumatologisk afdeling, Frederiksberg Hospital
[2] Røntgenafdelingen, Frederiksberg og Bispebjerg Hospitaler

STUDIESITE:
Parker Instituttet, Reumatologisk afdeling
Frederiksberg Hospital
Ndr. Fasanvej 57
2000 F, Danmark

DATO: Tirsdag, august 07, 2012

Version 1.2

Downloaded From: http://archinte.jamanetwork.com/ by a University of South Carolina User on 10/26/2015

INDHOLDSFORTEGNELSE

INDHOLDSFORTEGNELSE 2
KONTAKTOPLYSNINGER 4
UNDERSKRIFTSSIDE 5
PROTOKOL SYNOPSIS 6
FORKORTELSER 7
1. BAGGRUND 8
1.1 PRÆPARATNAVN OG BESKRIVELSE 8
1.2 VIRKNINGSMEKANISME 8
1.3 RESUMÉ AF KLINISKE FORSØG 8
1.4 DOSIS, DOSERINGSMÅDE OG -FREKVENS 8
1.5 KLINISK SIKKERHED OG EFFEKT 8
2. FORMÅL & HYPOTESE 9
2.1 FORSØGETS RATIONALE 9
2.2 FORMÅL 9
2.3 HYPOTESE 9
3. STUDIEDESIGN 10
3.1 PROJEKTBESKRIVELSE 10
3.2 RATIONALE FOR UNDERSØGELSENS DESIGN 10
3.3 UNDERSØGELSENS EFFEKTMÅL 10
3.4 RANDOMISERINGSMETODE, BLINDING, OG MINIMERING AF BIAS 12
3.5 STOPREGLER 13
4. FORSØGSDELTAGERE 13
4.1 REKRUTTERING AF FORSØGSDELTAGERE 13
4.2 INKLUSIONSKRITERIER 13
4.3 EKSKLUSIONSKRITERIER 13
5. INTERVENTIONER 14
5.1 INJEKTIONER 14
5.1.1 DOSERING OG ADMINISTRATION AF INJEKTIONER 14
5.2 TRÆNING 15
5.2.1 DOSERING OG AFVIKLING AF TRÆNINGEN 15
5.2.2 MONITORERING AF TRÆNINGSCOMPLIANCE 15
5.3 SAMTIDIG TRÆNING, MEDICINFORBRUG OG ØVRIG BEHANDLING 15
6. UNDERSØGELSENS EFFEKTMÅL 16
6.1 SIKKERHED 16
6.2 PRIMÆRT EFFEKTMÅL 16
6.3 SEKUNDÆRE EFFEKTMÅL 16
7. TIDSPLAN FOR UNDERSØGELSERNE 18
7.1 INKLUSION AF FORSØGSDELTAGERE 18
7.2 UNDERSØGELSER OG ØVRIG KONTAKT I LØBET AF PROJEKTET 18
7.3 SCREENING 18

Downloaded From: http://archinte.jamanetwork.com/ by a University of South Carolina User on 10/26/2015

7.4 UNDERSØGELSER 19
8. UDELUKKELSE FRA OG AFBRYDELSE AF PROJEKTET 20
8.1 UDELUKKELSE AF FORSØGSDELTAGERE 20
8.2 AFBRYDELSE AF PROJEKTET 20
9 STATISTISKE METODER 21
9.1 ANALYSEPOPULATIONEN 21
9.2 ANALYSE AF STUDIETS GENNEMFØRSEL 21
9.3 ANALYSE AF GRUPPESAMMENLIGNELIGHED 21
9.4 ANALYSE AF EFFEKT 21
9.5 MANGLENDE DATA 21
9.6 BESTEMMELSE AF SAMPLE SIZE 21
9.7 RANDOMISERINGSTEKNIK 21
10. SIKKERHEDSFORANSTALTNINGER OG PROCEDURER 22
10.1 BIVIRKNINGER OG UTILSIGTEDE HÆNDELSER 22
10.2 REGISTRERING AF BIVIRKNINGER OG UTILSIGTEDE HÆNDELSER 22
10.3 ANTIKONCEPTION 23
11 VIDENSKABSETISK REDEGØRELSE 23
11.1 GENERELLE OVERVEJELSER 23
11.2 PROCEDURER FOR AFGIVELSE AF DEN MUNDTLIGE INFORMATION 23
11.3 SKRIFTLIG INFORMATION 24
11.4 INFORMERET SAMTYKKE 24
11.6 VIDENSKABSETISK REDEGØRELSE FOR DE PLANLAGTE INTERVENTIONER 24
11.7 VIDENSKABSETISK REDEGØRELSE FOR DE PLANLAGTE MÅLEMETODER 24
11.8 VIDENSKABSETISK GODKENDELSE 25
12 OVERHOLDELSE AF LOVE OG REGLER 25
13. PROJEKTETS AFVIKLING 25
13.1 ADMINISTRATIV STRUKTUR 25
13.2 BEHANDLING AF FORTROLIGE OPLYSNINGER 25
13.3 ANMELDELSE TIL DATATILSYNET 26
13.4 ANMELDELSE TIL LÆGEMIDDELSTYRELSEN 26
13.5 ØKONOMISKE FORHOLD 26
13.6 INFORMATION TIL FORSØGSDELTAGERE 26
13.7 PUBLIKATION AF RESULTATER 26
13.8 KVALITETSKONTROL OG –SIKRING 26
13.9 FORSØGSDELTAGERFORSIKRING 27
13.10 TIDSPLAN 27
14. OVERSIGT OVER APPENDICES 27
15. REFERENCER 28

Downloaded From: http://archinte.jamanetwork.com/ by a University of South Carolina User on 10/26/2015

KONTAKTOPLYSNINGER

SPONSOR & FORSØGSLEDER:
Forsker Marius Henriksen, fysioterapeut, PhD
Laboratorium for Klinisk Bevægefunktion
Parker Instituttet, Reumatologisk afdeling
Frederiksberg Hospital
Ndr. Fasanvej 57
2000 F, Danmark

INVESTIGATOR & KLINISK ANSVARLIG LÆGE:
Institutchef Professor Henning Bliddal, dr. med.
Parker Instituttet, Reumatologisk afdeling
Frederiksberg Hospital
Ndr. Fasanvej 57
2000 F, Danmark

BIOSTATISTIKER:
Robin Christensen, MSc, PhD.
Parker Instituttet, Reumatologisk afdeling
Frederiksberg Hospital
Ndr. Fasanvej 57
2000 F, Danmark

MONITOR:
Københavns Universitetshospitals GCP-enhed
Bispebjerg Hospital, Bygning 51, 3.sal
Bispebjerg Bakke 23
2400 København NV, Danmark

MR-BILLEDDIAGNOSTIK:
Mikael Boesen, læge, PhD.
Røntgenafdelingen
Frederiksberg Hospital
Ndr. Fasanvej 57
2000 F, Danmark

Downloaded From: http://archinte.jamanetwork.com/ by a University of South Carolina User on 10/26/2015

UNDERSKRIFTSSIDE

Underskriften nedenfor udgør godkendelsen af denne protokol og dens bilag og skal sikre at forsøget udføres i overensstemmelse med protokollen, gældende dansk lovgivning og standarder for god klinisk praksis (GCP).
Jeg har læst og gennemgået forsøgsprotokollen grundigt. Idet jeg har læst og forstået de i forsøgsprotokollen indeholdte krav og betingelser, indvilliger jeg i at gennemføre det kliniske forsøg i henhold til internationale regler for god klinisk praksis og krav fra relevante myndigheder om kildedokument verificering og audit/inspektion af forsøget.
Jeg indvilliger i kun at benytte forsøgsmaterialet, herunder medicinen, i henhold til det i protokollen anførte.
Jeg er indforstået med, at ændringer i protokollen skal foretages i form af et amendment med forudgående skriftlig godkendelse fra sponsor.
Jeg er indforstået med, at overtrædelse af protokollen kan føre til afslutning af forsøget inden planlagt tid.

SPONSOR

Marius Henriksen
NAVN

DATO UNDERSKRIFT

INVESTIGATOR

Henning Bliddal
NAVN

DATO UNDERSKRIFT

Downloaded From: http://archinte.jamanetwork.com/ by a University of South Carolina User on 10/26/2015

PROTOKOL SYNOPSIS

Protokoltitel
KOMBINERET INTRAARTIKULÆR CORTICOSTEROID OG FYSIOTERAPEUTISK TRÆNING HOS PATIENTER MED KNÆARTROSE: ET RANDOMISERET KLINISK FORSØG

Studiepopulation
Patienter med knæartrose i alderen 40 år og opefter

Forsøgsintervention
Intraartikulær injektion af corticosteroid eller sterilt saltvand i afficeret knæ efterfulgt af fysioterapeutisk træning 30-60 min, 3 gange/uge i 12 uger, med opstart 2 uger efter injektion.

Forsøgsgrupper
- Intraartiklær injektion (knæ) af corticosteroid + efterfølgende fysioterapeutisk træning
- Intraartiklær injektion (knæ) af saltvand + efterfølgende fysioterapeutisk træning

Primært effektmål
- Ændringer i patientrapporteret smerte (Knee Injury and Osteoarthritis Outcome Score (KOOS) subskala: smerte) fra baseline til uge 14

Sekundære effektmål
- Ændringer i patientrapporteret smerte (KOOS subskala: smerte) fra baseline til uge 2 og 26
- Ændringer i patientrapporterede symptomer, funktion i dagligdagen, livskvalitet og funktion sport og fritid (KOOS subskalaer) fra baseline til uge 2, 14 og 26
- Ændringer i patientrapporteret funktion i hverdagsaktiviteter (ADL-Q spørgeskema) fra baseline til uge 2, 14 og 26
- Ændringer i tilstedeværelsen af neuropatisk smertekomponent (PainDetect spørgeskema) fra baseline til uge 2, 14 og 26
- Ændringer i funktionel smertetest fra baseline til uge 14 og 26
- Ændringer i smertefølsomhed (perifere og centrale smertemekanismer) fra baseline til uge 14 og 26
- Ændringer i inflammationsmarkører i blod, urin og ledvæske fra baseline til uge 14 og 26
- Ændringer i billeddiagnostiske inflammationsmarkører fra ultralyds- og MR-scanning fra baseline til uge 14 og 26
- Ændringer i biomekanisk ganganalyse fra baseline til uge 14 og 26

Eksplorative effektmål
- Ændringer i muskelstyrke fra baseline til uge 14 og 26
- Ændringer i fysisk funktionsniveau fra baseline til uge 14 og 26

Sample Size
50/gruppe = 100 deltagere i alt

Varighed
26 uger

Downloaded From: http://archinte.jamanetwork.com/ by a University of South Carolina User on 10/26/2015

FORKORTELSER

ADL-Q	Activities of daily living questionnaire
BMI	Bmody Mass Index
CRF	Case Report Form
EULAR	European League Against Rheumatism
GCP	Good Clinical Practice
ICH	International Conference on Harmonization
ITT	Intention To Treat
KOOS	Knee Osteoarthritis Outcome Score
MR	Magnetisk Resonans
OA	Osteoartrose
OARSI	OsteoArthritis Research Society International
SAD	Sygehusapotekerne i Danmark
VAS	Visuel Analog Skala

Downloaded From: http://archinte.jamanetwork.com/ by a University of South Carolina User on 10/26/2015

1. BAGGRUND

Denne undersøgelse er planlagt som en randomiseret, parallelarm, kontrolleret, dobbelt-blindet interventions-undersøgelse af effekten af kombineret intraartikulær injektion af corticosteriod og fysioterapeutisk træning af patienter med knæartrose (artrose: slidgigt, eng: osteoarthritis (OA)).

1.1 PRÆPARATNAVN OG BESKRIVELSE

Som aktivt stof anvendes Depo-Medrol® (40 mg, Pfizer) i form af injektionsvæske (suspension) iblandet 4 ml Lidokain (10 mg/ml, SAD).
Som komparator anvendes isotonisk natriumklorid (9 mg/ml, SAD) i form af injektionsvæske (suspension) iblandet 4 ml Lidokain (10 mg/ml, SAD).
Der henvises til Lægemiddelstyrelsens Produktresuméer (Depo-Medrol: Appendiks A, Lidokain: Appendiks B, natri-umklorid: Appendiks C) for nærmere beskrivelse af de anvendte præparater.

1.2 VIRKNINGSMEKANISME

Mekanismerne bag den lokale virkning af intraartikulær corticosteriod er ikke helt afklaret. En reduceret synovial perfusion og mikrovaskulær permeabilitet er formentlig af betydning (1). Den subjektive bedring er relateret til en øget smertetærskel, øget ledvæskeviskositet, nedsat ledtemperatur, nedsat ledvæskevolumen og nedsat synovialmembranvolumen (1).

1.3 RESUMÉ AF KLINISKE FORSØG

I en metaanalyse af effekten af intra-artikulær injektion af corticosteroid til knæ OA (2) inkluderedes randomiserede placebokontrollerede forsøg på patienter med knæartrose publiceret i perioden 1966-2003. I alt 35 studier blev identificeret hvoraf 10 studier opfyldte metaanalysens inklusionskriterer om høj metodisk kvalitet. I disse 10 højkvalitetsstudier var den poolede relative risiko for symptomforbedring 16-24 uger efter intraartikulær injektion af corticosteroid 2.09 (95% konfidensinterval 1.2 til 3.7), og number-needed-to-treat 4.4. Den poolede relative risiko for symptomforbedring i indtil 2 uger efter intraartikulær injektion af corticosteroid 1.66 (95% konfidensinterval 1.37 til 2.0). Number-needed-to-treat for at opnå forbedring hos én patient i studier med statistisk signifikante resultater var mellem 1,3 og 3,5 patienter.
De foreliggende kliniske studier understøtter såvel korttids (op til 2 uger) som langtids (16-24 uger) symptom-forbedring efter intraartikulær injektion af corticosteroid for knæ OA (2) .

1.4 DOSIS, DOSERINGSMÅDE OG -FREKVENS

I den aktive behandlingsarm injiceres 1 ml methylprednisolon corticosteroid (40 mg Depo-medrol®, Pfizer) iblandet 4 ml Lidokain (10 mg/ml, SAD). Dosis er bestemt ud fra vejledende dosering til brug i mellemstore og store led (1). Injektionen foretages ultralydsvejledt for at sikre korrekt afgivelse af injektionsbolus i synovial-sækken. I dette forsøg gives kun én dosis.

1.5 KLINISK SIKKERHED OG EFFEKT

Betydende systemiske bivirkninger ses ikke ved lokal injektionsbehandling. Rødme, varme og svedtendens opleves af op til 10% i minutter til timer (døgn) efter injektionen. Den alvorligste bivirkning er infektion af leddet, men ved aseptisk injektionsteknik ses infektion sjældnere end én gang pr. 10.000 injektioner. Hos 2% ses smertereaktion det første døgn efter injektion, måske som følge af synovialisirritation ved kontakt med de indførte mikrokrystaller. Synovialisirritation ses sjældent hos samme patient ved senere injektion.
Injektion af corticosteroider i led har en lokal antiinflammatorisk effekt med følgende smertelindring. Injektion af corticosteroider har været velkendt i mere end 50 år, med veldokumenteret effekt og sikkerhed (3;4).

Downloaded From: http://archinte.jamanetwork.com/ by a University of South Carolina User on 10/26/2015

1.6 RATIONALE FOR BEHANDLING

Intraartikulære injektioner af corticosteroider anbefales som behandling af knæ OA og betragtes i dag som standard medicinsk behandling af knæ OA. Intraartikulære injektioner af corticosteroider indgår således i de etablerede guidelines for behandling af knæ OA (5).

2. FORMÅL & HYPOTESE

2.1 FORSØGETS RATIONALE

Artrose i knæet er en yderst almindelig lidelse (6). Ikke-kirurgisk håndtering af patienter med knæ OA foretages typisk i et multidisciplinært regi, omfattende en kombination af non-farmakologiske og farmakologiske interventioner. Non-farmakologiske interventioner omfatter en lang række modaliteter, inklusiv fysioterapeutisk træning. De farmakologiske interventioner omfatter i første række smertelindring ved systemisk behandling med paracetamol, der ved utilfredsstillende effekt kan skiftes til non-steroide antiinflammatoriske lægemidler (NSAID). Sidstnævnte administreres med omhyggelig kontrol og opmærksomhed på bivirkninger (især cardiovaskulære, renale og gastrointestinale).
Lokal farmakologisk behandling af det syge knæled anbefales i form af intraartikulære injektioner af corticosteroider (binyrebarkhormon), der i dag betragtes som standard medicinsk behandling af knæ OA og således indgår i de etablerede guidelines for behandling af knæ OA (5). Injektion af corticosteroider har været velkendt i mere end 50 år, med veldokumenteret effekt og sikkerhed (4;7).
I den daglige håndtering af knæ OA henvises patienterne til fysioterapeut med henblik på træningsbehandling. Kliniske retningslinjer omfatter udover farmakologisk behandling også altid henvisning til fysioterapeutisk træning. Den 3. EULAR (European League Against Rheumatism) anbefaling anerkender den centrale vigtighed af træning (8), ligesom det internationale OA selskab (OsteoArthritis Research Society International; OARSI), også anbefaler træning som en central behandlingsform (5). Effektstørrelsen af træningsterapi er i den samme størrelsesorden som de farmakologiske behandlinger, men er oftest uden bivirkninger (5). Selvom den analgetiske mekanisme ved træning er ukendt, har eksperimentelle studier foreslået at træning eventuelt har en lokal antiinflammatorisk effekt (9;10).
Det er imidlertid hyppigt at patienterne henvises efter at have modtaget en intraartikulær farmakologisk behandling med corticosteroid. Dette vanskeliggør isoleret vurdering af træningseffekten.
Således eksisterer der mindst to standardregimer for ikke-kirurgisk behandling af patienter med knæ OA; 1) corticosteroid injektion + fysioterapeutisk træning og 2) fysioterapeutisk træning. Der foreligger imidlertid ingen studier der sammenligner de to standardregimer.

2.2 FORMÅL

Formålet med undersøgelsen er at undersøge effekten af fysioterapeutisk træning kombineret med farmakologisk inflammationshæmning på patientrapporterede smerter blandt patienter med knæ OA, samt at afdække mulige mekanismer i relation til denne effekt.

2.3 HYPOTESE

Det hypotetiseres at
Farmakologisk reduktion af lokal inflammation i knæet forud for en fysioterapeutisk træningsintervention forstærker de kliniske effekter af træningsterapien sammenlignet med en placebobehandling af lokal inflammation i knæet forud for en fysioterapeutisk træningsintervention.

Downloaded From: http://archinte.jamanetwork.com/ by a University of South Carolina User on 10/26/2015

3. STUDIEDESIGN

3.1 PROJEKTBESKRIVELSE

Dette er en randomiseret, parallelarm, kontrolleret, dobbelt-blindet interventionsundersøgelse af effekten af kombineret farmakologisk inflammationshæmning ved intraartikulær injektion af corticosteroid og fysioterapeutisk træning på patientrapporteret smerte og funktion, blandt patienter med knæ OA. Forsøgsdeltagerne vil blive randomiseret ligeligt (1:1) til 1 aktiv gruppe og 1 kontrolgruppe:

Forsøgsgrupper

- Intraartikulær injektion af corticosteroid efterfulgt af et fysioterapeutisk træningsforløb
- Intraartikulær injektion af sterilt isotont saltvand efterfulgt af et fysioterapeutisk træningsforløb

Forsøgsdeltagerne undersøges ved baseline (før randomisering), efter 14 uger (2 ugers virkningstid af corticosteroid + 12 ugers træning) og efter yderligere 12 uger (26 ugers follow-up). Træningen påbegyndes 2 uger (14 dage) efter injektionen.

3.2 RATIONALE FOR UNDERSØGELSENS DESIGN

Undersøgelsen er designet til at undersøge effekten af kombineret lokal corticosteroid behandling og fysioterapeutisk træning på patientrapporteret smerte og funktion blandt patienter med knæ OA. For at kunne vurdere virkningerne af kombinationen af lokal antiinflammatorisk behandling efterfulgt af fysioterapeutisk træning, er en kontrolgruppe inkluderet, der modtager træning efter en forudgående placeboinjektion (saltvand).

3.2.1 Rationale for træningsdosis og træningsvarighed

Træningsdoseringen (3 gange/uge) følger standardanbefalinger for terapeutisk træning til patienter med knæ OA. En træningsperiode på 12 ugers (84 dage) er valgt. De primære effektmål i undersøgelsen analyseres som den gennemsnitlige ændring i patientrapporterede smerter mellem Dag 0 (baseline) og Dag 98 (uge 14). Valget af træningsperiode er baseret på varigheden af en typisk træningsintervention i kliniske forsøg og fysioterapeutisk praksis. Observationsvarighed kan forlænges for enkelte personer på grund af bivirkninger, unormale resultater af laboratorietest, eller andre oplysninger, hvis det skønnes hensigtsmæssigt af den klinisk ansvarlige.

3.2.2 Rationale for observationsperiode

Det er valgt at opstarte træningen 2 uger efter injektionen, da den kliniske erfaring peger på at den maksimale effekt af intraartikulær behandling med corticosteroid opnås efter 2 uger.
Der er valgt 12 ugers (84 dage) træningsperiode, da dette er det hyppigst anvendte i såvel klinisk praksis som i videnskabelige undersøgelser af træning til patienter med knæ OA. Den gennemsnitlige virkningsvarighed af det anvendte corticosteroid angives at være mellem 8-56 dage (1), men virkningsvarigheden af kombinationen af lokal corticosteroidbehandling og træning er ukendt. Det er velkendt at de kliniske effekter af træning aftager gradvist i løbet af 3 måneder efter endt træningsprogram (11). For at undersøge om kombinationen af corticosteroid og fysioterapeutisk træning forlænger de kliniske effekter eller forsinker faldet i effekt, observeres patienterne også 12 uger efter endt træning (26 uger efter inklusion).

3.3 UNDERSØGELSENS EFFEKTMÅL

3.3.1 Primært effektmål

- Ændringer i patientrapporteret smerte (KOOS subskala: smerte (12)) fra baseline til uge 14

3.3.2 Sekundære Effektmål

- Ændringer i patientrapporteret smerte (KOOS subskala: smerte (12)) fra baseline til uge 2 og 26

Downloaded From: http://archinte.jamanetwork.com/ by a University of South Carolina User on 10/26/2015

- Ændringer i patientrapporterede symptomer, funktion i dagligdagen, livskvalitet og funktion i sport og fritid (KOOS subskalaer (12)) fra baseline til uge 2, 14 og 26
- Ændringer i patientrapporteret funktion i hverdagsaktiviteter (ADL-Q spørgeskema) fra baseline til uge 2, 14 og 26
- Ændringer i tilstedeværelsen af neuropatisk smertekomponent (PainDetect spørgeskema) fra baseline til uge 2, 14 og 26
- Ændringer i funktionel smertetest fra baseline til uge 2, 14 og 26
- Ændringer i smertefølsomhed (perifere og centrale smertemekanismer) fra baseline til uge 14 og 26
- Ændringer i inflammationsmarkører fra blod, urin og ledvæske fra baseline til uge 14 og 26
- Ændringer i ultralydsbedømt inflammation fra baseline til uge 14 og 26
- Ændringer i inflammation bedømt ved kontrastforstærket MR fra baseline til uge 14 og 26.
- Ændringer i biomekanisk ganganalyse fra baseline til uge 14 og 26

3.3.3 Eksplorative Effektmål

- Ændringer i muskelstyrke fra baseline til uge 14 og 26
- Ændringer i fysisk funktionsniveau fra baseline til uge 14 og 26

3.3.4 RATIONALE FOR UNDERSØGELSENS EFFEKTMÅL

Patientrapporteret smerte (primært effektmål)
For at undersøge behandlingseffekten på patientens oplevelse af smerter anvendes subskalaen 'smerter' fra spørgeskemaet Knee Injury and Osteoarthritis Outcome Score (KOOS). KOOS spørgeskemaet er et validt instrument til registrering af patienternes vurderinger af smerter.

Patientrapporterede symptomer, funktion i dagligdagen, livskvalitet og funktion i sport og fritid (sekundære effektmål)
For at undersøge behandlingseffekten på patientens oplevelse af symptomer, funktion i dagligdagen, livskvalitet og funktion i sport og fritid anvendes subskalaerne 'symptomer', 'funktion i dagligdagen', 'livskvalitet', og 'funktion i sport og frtitid' fra spørgeskemaet Knee Injury and Osteoarthritis Outcome Score (KOOS). KOOS spørgeskemaet er et validt instrument til registrering af patienternes vurderinger af symptomer, funktion i dagligdagen, livskvalitet og funktion i sport og fritid.

Patientrapporteret funktion i hverdagsaktiviteter (sekundært effektmål)
For at undersøge patientens oplevelse af behandlingseffekten på aktivitetsformåen indenfor en række hverdagsaktiviteter, anvendes spørgeskemaet ADL-taxonomi (ADL-Q). ADL-Q er et validt instrument til registrering af patienternes vurderinger af aktivitetsformåen.

Tilstedeværelsen af neuropatisk smertekomponent
For at undersøge behandlingseffekten på tilstedeværelse af neuropatisk smertekomponent, anvendes spørgeskemaet PainDetect. PainDetect er et validt instrument til vurdering af tilstedeværelsen af neuropatisk smertekomponenter.

Funktionel smertetest
Bevægelse er ofte er forbundet med smerte hos patienter med kroniske smerter, enten ved igangsætning, efter en vis tid/distance, eller efterfølgende. Derfor registreres smerte og smerteudvikling under standardiserede bevægelser, som et mål for smerte under fysisk funktion.

Downloaded From: http://archinte.jamanetwork.com/ by a University of South Carolina User on 10/26/2015

Smertefølsomhed og perifere og centrale smertemekanismer
Ændringer i smertefølsomhed kan være forårsaget af ændringer i følsomheden af nociceptorer beliggende i dybe væv (perifer sensibilisering eller de-sensibilisering) eller som ændringer i det centralnervøse respons (central sensibilisering eller de-sensibilisering). Ændringerne er relateret til ændret synaptisk aktivitet i spinale neuroner og ændringer i descenderende kontrol fra supraspinale centre. Tryksmertetærskler er anvendelige til at vurdere perifer sensibilisering i knæ OA (13). Manifestationer af forskellige aspekter af sensibilisering vurderes kvantitativt ved hjælp af trykalgometri (14). Computerstyret manchet-algometri med en passende timing af stimulationsparametre bruges til at detektere faciliterede centrale smertemekanismer (fx temporale og spatiale summations-fænomener) som angivelse af central sensibilisering (15).

Proinflammatoriske cytokiner i blod, urin og ledvæske
For at vurdere ændringen i lokal inflammation aspireres ledvæske der analyseres for koncentrationer af proinflammatoriske cytokiner. Ledvæsken aspireres ultralydsvejledt.
For at vurdere ændringer i systemisk koncentration af brusknedbrydningsstoffer og inflammationsstoffer tages en blodprøve samt en urinprøve.

Billeddiagnostiske inflammationsmarkører fra ultralyds-scanning
For at undersøge graden og udbredelsen af inflammation i knæet gennemføres ultralydsscanninger af forsøgsdeltagernes knæ.

Billeddiagnostiske inflammationsmarkører fra MR-scanning
For at vurdere den strukturelle status og udbredelsen af inflammation i knæet gennemføres MR skanninger af forsøgsdeltagernes knæ med flere forskellige MR optagelser før og efter indgivelse af intravenøs Gadolinoium-kontrast. Kontrast gives kun til patienter med forudgående normal nyrefunktion bedømt ved blodprøve og estimeret Glomeruloinfiltrations rate der skal være over 0.60.

Biomekanisk ganganalyse (sekundært effektmål)
Biomekaniske faktorer er af væsentlig betydning for knæets funktion og patientens mobilitet. Disse kvantificeres ved en biomekanisk ganganalyse.

Muskelstyrke og fysisk funktionsniveau (eksplorative effektmål)
For at dokumentere at træningen har været på en tilpas høj intensitet og derved medfører muskelstyrketilvækst hhv. øget fysisk funktionsniveau, måles dette.

3.4 RANDOMISERINGSMETODE, BLINDING, OG MINIMERING AF BIAS

3.4.1 Metode til Gruppetildeling og Randomisering

Parker Instituttets statistiker vil være ansvarlig for udvikling af randomiseringsliste inden projektets start. Randomiseringen foretages i permuterede blokke á 2-6 for at tage højde for eventuelle sæsonvariationer. Randomiseringen foretages 1:1, med 50 deltagere i hver gruppe. Et duplikat af randomiseringslisten leveres til og opbevares af forskningsadministrationen på Parker Instituttet.
Efter inklusion af en forsøgsdeltager, indhentning af underskrevet informeret samtykke, og baselinemålinger, vil den kliniske farmaceut tildele forsøgsdeltageren et randomiseringsnummer fra randomiseringslisten (se afsnit 9.7).

Downloaded From: http://archinte.jamanetwork.com/ by a University of South Carolina User on 10/26/2015

3.4.2 Blinding

Dette er en dobbelt-blindet, kontrolleret undersøgelse. Patienter, forskere, studiekoordinatorer, klinisk personale, projektmedarbejdere, forskningsmedarbejdere, datahåndteringspersonale, og andet personale som er direkte involveret i undersøgelsen, vil blive blindet overfor injektionstype.
Oplysninger med potentiale til at afblinde blindet personale vil ikke blive videregivet og vil blive opbevaret aflåst med begrænset adgang, indtil afslutningen af forsøget.
Hvis investigator finder det nødvendigt, kan injektionstype afblindes umiddelbart, og det er investigators suveræne beslutning. Afblindingen foregår ved at investigator kontakter Parker Instituttets forskningsadministration og modtage information om hvilken injektion patienten har modtaget (corticosteroid eller saltvand) ved at oplyse randomiseringsnummeret, hvorefter randomiseringskoden brydes og injektionstypen oplyses af Parker Instituttets forskningsadministration. Når afblinding er foretaget for en patient kan denne ikke fortsætte deltagelse i den fysioterapeutiske træning.

3.5 STOPREGLER

Projektet skal afbrydes hvis der opstår forhold der kompromitterer forsøgsdeltagernes sikkerhed eller udgør en sundhedsrisiko.
Ved mistanke herom skal projektet i første omgang suspenderes mens forholdet undersøges nærmere, og det kan afgøres om forholdet kan henføres til interventioner og/eller undersøgelser der indgår i projektet.

4. FORSØGSDELTAGERE

4.1 REKRUTTERING AF FORSØGSDELTAGERE

Patienter rekrutteres fra Frederiksberg Hospitals reumatologiske ambulatorium, og via annoncer i dag- og ugeblade, opslag på opslagstavler, hjemmesider o.l.

4.2 INKLUSIONSKRITERIER

Forsøgsdeltagerne skal opfylde følgende inklusionskriterier for at kunne deltage i undersøgelsen:

- Alder over 40 år
- Diagnosticeret knæartrose jf. diagnostiske kriterier
- Radiologisk verificerede forandringer foreneligt med tibiofemoral artrose
- Smerter svarende til mindst 40 mm på en 0-100 mm VAS på spørgsmålet ”smerte ved gang på flad vej”
- Kliniske tegn på lokal inflammation i knæet
- Villig til at stoppe brug af smertestillende medicin mens undersøgelsen pågår
- Villig til og mulighed for at deltage i alle undersøgelser
- Villig til og mulighed for at deltage i alle træningssessioner
- 20≤ Body Mass Index (BMI) ≤35 kg/m^2
- Villig til at overholde regler om samtidig medicinering og behandling (se afsnit 5.4)
- Taler, læser og skriver Dansk

4.3 EKSKLUSIONSKRITERIER

Personer der opfylder følgende eksklusionskriterier kan ikke deltage i undersøgelsen:

- Deltagelse i fysioterapeutisk træning for knæartrose indenfor de seneste 3 måneder
- Behandling med intraartikulær corticosteroid i knæ indenfor de seneste 3 måneder
- Kontraindikationer mod træning
- Kontraindikationer mod intraartikulær corticosteroid injektion

Downloaded From: http://archinte.jamanetwork.com/ by a University of South Carolina User on 10/26/2015

- Gravid eller ammende
- Nuværende eller tidligere symptomer på autoimmun sygdom (fx, inflammatoriske tarmlidelser, multipel sclerose, lupus, rheumatoid arthrit)
- Planlagte kirurgiske indgreb under studieperioden
- Nuværende eller tidligere diagnose, tegn eller symptomer på betydelig kardiovaskulære sygdom, inklusiv, men ikke begrænset til:
    - Iskæmisk hjertesygdom
    - Arterosclerose
- Medicinske forhold der kontraindicerer træning, inklusiv, men ikke begrænset til:
    - Kronisk eller medfødt hjertesygdom
    - Svær astma
    - Kronisk obstruktiv lungesygdom
- Tidligere eller nuværende diagnose, tegn eller symptomer på betydelig neurologisk sygdom, inklusiv, men ikke begrænset til:
    - Blodprop i hjernen
    - Hjerneblødning
    - Klinisk betydeligt hovedtraume inden for det sidste år
    - Perifer neuropati
    - Epilepsi eller krampeanfald
    - Svært nedsat balance
- Alkohol- eller narkotikamisbrug inden for de seneste 5 år
- Tidligere eller nuværende diagnose, tegn eller symptomer på betydelig psykiatrisk lidelse
- Smertesyndromer som fx fibromyalgi
- Regionale smerter forårsaget af lumbalt eller cervicalt nerverodstryk med radiculopati eller med risiko for udvikling heraf
- Et hvilket som helst andet forhold der efter investigators mening vil udsætte en potential forsøgsdeltager i øget sikkerhedsrisiko eller på anden vis gør personen uegnet til deltagelse i projektet

5. INTERVENTIONER

5.1 INJEKTIONER

Intraartikulær corticosteroidinjektion (COR):
Injektionerne foretages under ultralydsvejledning. Inden injektion aspireres betydelig effusion (der gemmes til senere analyse, se afsnit 6.2.4) hvorefter der injiceres 1 ml methylprednisolon corticosteroid (40 mg Depo-Medrol®) iblandet 4 ml Lidokain (10 mg/ml, SAD) uden adrenalin.

Intraartikulær saltvandsinjektion (SAL):
Injektionerne foretages under ultralydsvejledning. Inden injektion aspireres betydelig effusion (der gemmes til senere analyse, se afsnit 6.2.4) hvorefter der injiceres 1 ml isotont saltvand iblandet 4 ml Lidokain (10 mg/ml, SAD) uden adrenalin.

5.1.1 DOSERING OG ADMINISTRATION AF INJEKTIONER

Afvikling
For at sikre blinding af både forsøgsdeltager og den person der udfører injektionen foretages præparationen af injektionssprøjterne af Parker Instituttets kliniske farmaceut, jf. randomiseringen (COR eller SAL se afsnit 5.1),

Downloaded From: http://archinte.jamanetwork.com/ by a University of South Carolina User on 10/26/2015

uden tilstedeværelse af blindede personer (herunder forsøgsdeltageren og den person der udfører injektionen).
Da corticosteroidopløsningen (COR) er mælkehvid og saltvand (SAL) er gennemsigtigt, omvikles injektionssprøjterne efter iblanding med vehicle (4 ml 1% lidokain) med uigennemsigtigt tape for ikke at afsløre indholdets farve. Herefter overdrages sprøjten til den person der udfører injektionen. Sprøjten vil være forsynet med randomiseringskode.

5.2 TRÆNING

Fysioterapeutisk træning:
Den fysioterapeutiske træning omfatter styrke-, og koordinationstræning. Før træningen gennemføres en 10-minutters opvarmningsfase på en kondicykel ved moderat intensitet. Styrketræningen foregår både i standard styrketræningsmaskiner samt ved funktionelle øvelser med frie vægte, elastikker og/eller kropsvægt som modstand. Belastningen justeres med afsæt i både forbedringer og forværringer i såvel funktion, smerter og træningskvalitet.
Træningen superviseres af en fysioterapeut. Belastning, antal sæt og repetitioner noteres i en personlig træningsdagbog for hver deltager.
Der er planlagt alternativ ”rescue” træning til situationer hvor en deltager har smerter eller andre forhold relateret til deres sygdom, som forhindrer gennemførelse af det fulde træningsprogram.
Træningsprogrammet er beskrevet i særskilt dokument.

5.2.1 DOSERING OG AFVIKLING AF TRÆNINGEN

Afvikling
Den fysioterapeutiske foregår i fysioterapiafdelingen på Frederiksberg Hospital. Træningen påbegyndes 2 uger (14 dage) efter injektion.

Dosering
Intervention fra Dag 15 til Dag 98 (inklusiv): 3 sessioner per uge.

Dosismodifikation
Dosismodifikationer er tilladte ved at ændre træningsintensiteten (jf. afsnit 5.2). Ingen ændringer i træningsfrekvensen (antal sessioner per uge) er tilladt.

5.2.2 MONITORERING AF TRÆNINGSCOMPLIANCE

Antal gennemførte træningsssessioner registreres. Forsøgsdeltagerens og fysioterapeutens vurdering af træningsintensitet registreres ved hvert besøg.

5.3 SAMTIDIG TRÆNING, MEDICINFORBRUG OG ØVRIG BEHANDLING

Ingen nye træningsregimer må påbegyndes under forsøgsdeltagelsen. Anden fysioterapi, massage, elstimulation (fx TENS) eller alternative behandlinger (fx akupunktur) er ikke tilladte. Varme/kulde terapi må anvendes, undtagen inden for 2 timer inden en træningssession og 24 timer inden en undersøgelse.
Smertestillende medicin er tilladt for mindre forhold, som fx hovedpine og træningsinduceret ømhed. Anvendt medicin registreres i træningsdagbogen. Ingen smertestillende medicin er tilladt 24 timer eller 5 halveringstider (alt efter hvilken der er længst) forud for en undersøgelse.

Downloaded From: http://archinte.jamanetwork.com/ by a University of South Carolina User on 10/26/2015

5.3.1 Forbudte præparater og behandlinger

De følgende præparater er ikke tilladte at anvende under projektet.

- Systemiske corticosteroider
- TNF-α hæmmere
- Opioider
- Lejlighedsvis brug af sedativer eller hypnotica (fx lorazapam, temezapam, triazolam, alprazolam, zolpidem, chlordiazepoxide) er tilladt som sovemedicin, dog ikke 3 dage forud for en undersøgelse. Præparaterne må ikke anvendes som muskelrelaxanter.
- Ingen eksperimentelle behandlinger (medicinsk som fysikalsk) er tilladte under projektdeltagelsen

Under projektdeltagelsen instrueres deltagerne i at oplyse ny receptpligtig eller håndkøbsmedicin til den klinisk ansvarlige eller projektlederen. Al medicinbrug noteres på deltagerens case report form (CRF).

6. UNDERSØGELSENS EFFEKTMÅL

De undersøgelser der indgår i dette projekt beskrives i det følgende. Tidsplan præsenteres i afsnit 7 og Appendiks D.

6.1 SIKKERHED

Før og efter hver træningsgang vurderer deltageren sin smerte på en numerisk rangskala (0-10). Der er planlagt alternativ ”rescue” træning til situationer hvor en deltager har smerter eller andre forhold som forhindrer gennemførelse af det fulde træningsprogram.

6.2 PRIMÆRT EFFEKTMÅL

6.2.1 Patientrapporteret smerte

Patienternes egen opfattelse af smerter i relation til deres knæsygdom registreres vha. subskalaen ’smerter’ i spørgeskemaet ’Knee Osteoarthritis Outcome Score’ (KOOS) (12), vedlagt denne protokol. Der anvendes dansk udgave af spørgeskemaet der er valideret overfor det originale.

6.3 SEKUNDÆRE EFFEKTMÅL

6.3.1 Patientrapporterede knærelaterede symptomer, funktion i dagligdagen, livskvalitet og funktion i sport og fritid

Patienternes egen opfattelse af symptomer, funktion i dagligdagen, livskvalitet og funktion i sport og fritid i relation til deres knæsygdom registreres vha. subskalaerne ’symptomer’, ’funktion i dagligdagen’, ’livskvalitet’ og ’funktion i sport og fritid’ i spørgeskemaet ’Knee Osteoarthritis Outcome Score’ (KOOS) (12), vedlagt denne protokol. Der anvendes en dansk udgave af spørgeskemaet, der er valideret overfor det originale.

6.3.2 Patientrapporteret funktion i hverdagsaktiviteter

Til at dokumentere patienternes aktivitetsformåen indenfor en række hverdagsaktiviteter anvendes spørgeskemaversionen af ADL Taxonomien, ADL-Q (16), vedlagt denne protokol. Der anvendes en dansk udgave af spørgeskemaet, der er valideret overfor det originale.

6.3.3 Tilstedeværelse af neuropatisk smertekomponent

Til kortlæggelse af omfanget af neuropatiske smertetilstande anvendes spørgeskemeat ’PainDetect’ (17), vedlagt denne protokol. Der anvendes en dansk udgave af spørgeskemaet, der er valideret overfor det originale.

Downloaded From: http://archinte.jamanetwork.com/ by a University of South Carolina User on 10/26/2015

6.3.4 Funktionel smertetest

Patienternes knæsmerter registreres under standardiserede fysiske funktionstest. De fysiske funktionstest omfatter bl.a. gang, rejse-sætte-sig, trappegang. Smerteintensiteten i indeksknæet registreres ved teststart og løbende under testen på en numerisk rangskala (0-10). Patienten kan afbryde testen undervejs som følge af træthed og/eller smerter. Tidsvægtede gennemsnit af smerteintensiteten er resultatet. Testene superviseres af en fysioterapeut.

6.3.5 Smertefølsomhed

Computer-kontrolleret manchet trykalgometri

En lufttryksmanchet fastgøres på underbenet, på det sted hvor muskelbugen er tykkest. En computerstyret kompressor pumper via en slange luft i manchetten med en konstant hastighed, hvilket bevirker en langsom trykstigning. Forsøgspersonen vil først føle, at det klemmer sammen om benet, hvorefter det vil begynde at gøre ondt. Forsøgsdeltagerne markerer tidspunktet for tryksmertetærsklen via en trykknap, hvorefter stimulationen afbrydes. Længerevarende trykstimulering (6 min.) kan føre til en forøgelse af smerteoplevelsen (temporal summation). Denne metode er anvendelig til bestemmelse af hvorvidt smertesensitiviteten er forandret af perifere eller centrale mekanismer da temporal summation er centralt betinget (18). Hvis forsøgspersonen føler et sådant ubehag, at forsøgspersonen ikke vil fortsætte, kan forsøgspersonen afbryde målingen på en stop-knap inden de 6 minutter er gået. Det computer-kontrollerede manchet trykalgometer er udviklet til bestemmelse af følsomhed og smertefølsomhed i muskler og dybt væv (19). Målinger foretages unilateralt på det involverede ben.

6.3.6 Inflammationsmarkører fra blod, urin og ledvæske

Der udføres ultralydsvejledt aspiration af ledvæske fra involveret knæ (knæet tømmes så mængden kan ikke angives). Forsøgsdeltagerne møder fastende til blodprøvetagning og aflevering af urinprøver (minimum 22,5 ml per gang i alt 45 ml). Blodprøve (22,5 ml) trækkes fra regio cubitis ifølge Frederiksberg Hospitals standardprocedurer for blodprøvetagning. Til opbevaring af ledvæske, blod- og urinprøver oprettes en forskningsbiobank. Formålet med biobanken er at opbevare det biologiske materiale under sikre og kontrollerede forhold indtil en samlet analyse af alt det indsamlede materiale kan foretages. Dette gøres af økonomiske og praktiske årsager. Materialet gemmes indtil endt analyse (i maksimalt 5 år) og destrueres derefter. Ledvæske, urin- og blodprøver analyseres i eget laboratorium og residualer destrueres efter endt analyse.

6.3.7 Ultralydsvurdering af inflammation

Ultralydsundersøgelsen omfatter vurdering af bløddele, menisker og synovial hypertrofi og perfusion.

Ultralydsundersøgelsen foretages med en General Electric Logiq9™ scanner. Undersøgelsen foregår med forsøgsdeltageren liggende på et undersøgelsesleje.

6.3.8 Billeddiagnostiske inflammationsmarkører fra MR-scanning

Der anvendes en MR protokol der bruges til at give information om brusk, knogle og omkringliggende strukturer. Der anvendes en Siemens 3Tesla Verio scanner. Der scannes før, under og efter intravenøs injektion af kontraststof (singledose Gadolinium kontrast, 0.05mmol, Prohance, Bracco International, Holland). Der injiceres 0,1 ml/kg kropsvægt med en power injector (2ml per sekund). Total skannings tid 30-40 min. Scanningerne vil blive bedømt og scoret for strukturel ledskade ved brug af semikvantitative metoder (20) og de dynamiske MR data til kvantificering af betændelsesgraden vil blive analyseret vha. softwaret Dynamika®(21;22). Detaljer om MR-protokollen findes i et særskilt dokument.

6.3.9 Biomekanisk ganganalyse

Downloaded From: http://archinte.jamanetwork.com/ by a University of South Carolina User on 10/26/2015

Gangmønsteret måles i et ganglaboratorium ifølge internationale standarder (23). Ganganalysen foretages med sko. Ved en ganganalyse måles gangbevægelsen vha. specialudviklede kameraer (6 stk.) med samtidig måling af underlagsreaktionskræfter vha. kraftplatforme monteret i gulvet. Data integreres med antropometriske mål i en proces kaldet *inverse dynamics* (23;24) der resulterer i 3-dimensionel kinematik (hastighed, ledvinkler m.m.) og kinetik (kræfter, momenter m.m.) for led og muskelgrupper omkring ankel, knæ og hofte.

6.3.10 Muskelstyrke

Den maksimale muskelstyrke måles unilateralt vha. et isokinetisk dynamometer. Forsøgsdeltagerne placeres komfortabelt i dynamometerstolen, og dynamometerets rotationsakse justeres så den sammenfalder med ledrotationsaksen (fleksion/ekstension). Den distale del af deltagerens ben fastgøres til dynamometerets arm med en manchet. Forsøgsdeltagerne instrueres i at ekstendere og flektere knæet med maksimal muskelkraft. Muskelstyrken måles isometrisk (0 grader/sek.) ved 60 graders knæfleksion. Der gennemføres i alt tre målinger med en kort pause imellem.

6.3.11 Fysisk funktionsniveau

Seks minutters gangtest bruges som et mål for det almene fysiske funktionsniveau (25). Testens resultat vurderer både kredsløbsfunktionen og bevægeapparatets funktion som helhed. Forsøgsdeltageren bedes om at gå en prædefineret rute på 100 m så mange gange som muligt på 6 minutter. Resultatet er den tilbagelagte afstand i meter.

7. TIDSPLAN FOR UNDERSØGELSERNE

Hver undersøgelse skal foregå i henhold til det skemalagte undersøgelsestidspunkt (se Appendiks D).

7.1 INKLUSION AF FORSØGSDELTAGERE

Inklusion defineres som opfyldelse af inklusionskriterier og udelukkelse af eksklusionskriterier ved dag 1 samt komplette baselinemålinger.

7.2 UNDERSØGELSER OG ØVRIG KONTAKT I LØBET AF PROJEKTET

Efter inklusion og baselineundersøgelser, skal forsøgsdeltagerne møde til undersøgelse to gange. Forsøgsdeltagerne vil i løbet af projektet modtage telefonopringninger af forsøgsleder/studiekoordinator med henblik på afklaring af eventuelle projektrelaterede spørgsmål, og generel opfølgning på deltagelse i projektet. Forsøgsdeltagerne kan derudover kontakte forsøgsleder og studiekoordinator per telefon eller mail ved behov. Opstår der bivirkninger eller risiko for forsøgsdeltagernes sikkerhed og/eller sundhed i forbindelse med deltagelse i projektet, kan ekstraordinære undersøgelser blive planlagt.

7.3 SCREENING

Før screeningsundersøgelser må potentielle forsøgsdeltagere frivilligt underskrive en informeret samtykkeerklæring gældende for deltagelse i undersøgelsen.

7.3.1 Screeningsbesøg (Dag -30 til -1)

Ved screeningsbesøg foregår følgende aktiviteter:

- Frivillig afgivelse af informeret samtykke i form af underskrift
- Vurdering af deltager mht. opfyldelse af in- og eksklusionskriterier
- Demografiske oplysninger (køn, alder, højde, vægt m.m.)
- Medicinsk og fysikalsk anamnese

Downloaded From: http://archinte.jamanetwork.com/ by a University of South Carolina User on 10/26/2015

- Gennemgang og notering af medicinforbrug og træning de seneste 6 måneder
- Lægeundersøgelse
- Hvis nødvendigt, parakliniske undersøgelser (fx blodprøver, røntgen, EKG)

7.4 UNDERSØGELSER

Alle undersøgelser foregår på Parker Instituttet eller tilknyttede afdelinger på Frederiksberg Hospital. I Appendiks D findes en skematisk oversigt over undersøgelserne.

7.4.1 Dag 1 (Baseline)

- Vurdering af deltager mht. opfyldelse af in- og eksklusionskriterier

Hvis en potentiel deltager opfylder alle inklusionskriterier og ingen eksklusionskriterier:

- Bestemmelse af indeks knæ defineret som det symptomatiske knæ. Hvis bilateral involvering: Det mest symptomgivende knæ i løbet af den sidste måned.
- Baselineundersøgelser:
- Udfyldelse af spørgeskemaer
- Funktionel smertetest
- Undersøgelse af smertefølsomhed
- Biomekanisk ganganalyse
- Estimering af fysisk funktionsniveau
- Måling af muskelstyrke
- Blodprøve
- Urinprøve
- MR-scanning
- Ultralydsundersøgelse
- Aspiration af ledvæske
- Randomisering
- Injektion

7.4.2 Dag 15

- Træning påbegyndes
- Udfyldelse af spørgeskemaer

7.4.3 Dag 99 (14 uger)

Undersøgelser:

- Udfyldelse af spørgeskemaer
- Funktionel smertetest
- Undersøgelse af smertefølsomhed
- Biomekanisk ganganalyse
- Estimering af fysisk funktionsniveau
- Måling af muskelstyrke
- Blodprøve
- Urinprøve
- MR-scanning
- Ultralydsundersøgelse

Downloaded From: http://archinte.jamanetwork.com/ by a University of South Carolina User on 10/26/2015

- Aspiration af ledvæske

7.4.4 Dag 182

Undersøgelser:

- Udfyldelse af spørgeskemaer
- Funktionel smertetest
- Undersøgelse af smertefølsomhed
- Biomekanisk ganganalyse
- Estimering af fysisk funktionsniveau
- Måling af muskelstyrke
- Blodprøve
- Urinprøve
- MR-scanning
- Ultralydsundersøgelse
- Aspiration af ledvæske

Afslutning og tak for deltagelse

Note: Hvis en forsøgsdeltager præsenterer sig med en opstået anormalitet ved de afsluttende undersøgelser eller en vedblivende bivirkning til træningen, følges deltageren indtil normalisering af forholdet eller til forholdet kan beskrives som værende stabilt eller kronisk.

7.4.5 Afslutning af forsøget

Forsøget betragtes afsluttet, når den sidste forsøgsdeltager har gennemført det sidste follow-up besøg på Dag 182.

For hver enkelt forsøgsdeltager vil forsøget betragtes som afsluttet, efter hans/hendes sidste undersøgelser 182 dage efter inklusion i forsøget.

8. UDELUKKELSE FRA OG AFBRYDELSE AF PROJEKTET

8.1 UDELUKKELSE AF FORSØGSDELTAGERE

8.1.1 Årsager til individuel udelukkelse af forsøgsdeltagere

Forsøgsdeltagere må tilbagetrække deres informerede samtykke på et hvilket som helst tidspunkt.

Følgende kriterier for individuel udelukkelse/afbrydelse omfatter, men er ikke begrænset til:

- Et hvilken som helst medicinsk forhold – inklusiv bivirkninger til træningen – der efter den klinisk ansvarlig læges eller projektlederens mening udgør en sikkerheds- eller sundhedsrisiko for den enkelte forsøgsdeltager
- Manglende fremmøde til træning (mere end 9 træningssessioner) og/eller undersøgelser.

8.2 AFBRYDELSE AF PROJEKTET

Sponsor kan på et hvert tidspunkt afbryde projektet. Årsagerne hertil omfatter, men er ikke begrænset til:

- Forekomster eller hyppighed af alvorlige bivirkninger i dette eller andre undersøgelser, som viser en potentiel sundhedsfare for deltagerne
- Utilfredsstillende rekruttering af forsøgsdeltagere

Downloaded From: http://archinte.jamanetwork.com/ by a University of South Carolina User on 10/26/2015

9 STATISTISKE METODER

9.1 ANALYSEPOPULATIONEN

Effektanalyser foretages på intention-to-treat (ITT) populationen, defineret som forsøgspersoner der har fuldført baseline undersøgelser.

9.2 ANALYSE AF STUDIETS GENNEMFØRSEL

Forsøgsdeltagere der udelukkes af undersøgelsen opgøres per gruppe sammen med årsager til udelukkelse.

9.3 ANALYSE AF GRUPPESAMMENLIGNELIGHED

Demografiske variable og baseline karakteristika såsom alder, køn, vægt og BMI samt prognostiske faktorer såsom resultater fra baselinemålinger opgøres og summeres for hver gruppe. Middelværdier, standard deviationer, medianer, og ranges anvendes for kontinuerte variable. Antal og proportioner anvendes for kategoriske variable.

9.4 ANALYSE AF EFFEKT

Alle effektanalyser foretages på intention-to-treat (ITT) populationen. Alle statistiske tests er 2-halede med alpha = 0,05.

9.4.1 Primært Effektmål

Ændringer fra baseline til uge 14 sammenlignes mellem grupperne. Der anvendes repeated measures analyse med justering for baselineværdien. Eksplorative analyser udføres også med udvalgte demografiske og baseline variable som covariater for at undersøge effekten af øvrige faktorer på det primære effektmål.

9.4.2 Sekundære Effektmål

Ændringer fra baseline til uge 2 (kun spørgeskemaer), 14 og 26 sammenlignes mellem grupperne. Der anvendes repeated measures analyse med justering for baselineværdien.

9.5 MANGLENDE DATA

Manglende data erstattes ved multiple imputation.

9.6 BESTEMMELSE AF SAMPLE SIZE

Ved brug af en to-gruppe sammenlignende t-test, med et to-sidet statistisk signifikansniveau på P=0.05, og en forudsætning om en standard afvigelse (SD) på 15 point på primært effektmål (KOOS-smerte subskala), vil 50 patienter i hver gruppe give tilstrækkelig statistisk styrke (power>90%) til at kunne detektere en gruppe forskel på 10 point.

9.7 RANDOMISERINGSTEKNIK

Efter baselinemålinger randomiseres forsøgsdeltagerne til COR eller SAL grupperne ved kuvertbaseret randomisering. Før projektets start oprettes en mappe indeholdende 100 kuverter, hver med et stykke papir i, hvorpå der enten står ”COR” eller ”SAL”. Rækkefølgen af indholdet i kuverterne modsvarer randomiseringslisten der er udarbejdet af projektets statistiker (se afsnit 3.4.1). Det sikres at konvolutterne er lukkede og uigennemsigtige. Kuverterne nummereres konsekutivt fra 1-100 og placeres i mappen jf. denne nummerering (med nr. 1 forrest og nr. 100 bagerst). Kuverternes nummer er lig med randomiseringsnummeret. Mappen opbevares i et aflåst skab på den kliniske farmaceuts kontor. Et duplikat af randomiseringsliste og kuverter leveres til og op-

Downloaded From: http://archinte.jamanetwork.com/ by a University of South Carolina User on 10/26/2015

bevares af forskningsadministrationen på Parker Instituttet.
Når en forsøgsdeltager har gennemført baselinemålingerne, trækker den ublindede kliniske farmaceut en kuvert fra mappen startende med den forreste. Kuverten påtegnes forsøgsdeltagerens navn, fødselsdato og screeningsnummer samt dato for indrullering i projektet.
Efter randomisering foretages præparationen af injektionssprøjterne af den kliniske farmaceut, jf. randomiseringen (COR eller SAL se afsnit 5.1), uden tilstedeværelse af blindede personer (herunder forsøgsdeltageren og den person der udfører injektionen). Batch-numre for de anvendte lægemidler noteres og ilægges kuverten.
Da corticosteroidopløsningen (COR) er mælkehvid og saltvand (SAL) er gennemsigtigt, omvikles injektionssprøjterne efter iblanding med vehicle (4 ml 1% Lidokain) med uigennemsigtigt tape for ikke at afsløre indholdets farve. Herefter overdrages sprøjten til den person der udfører injektionen. Sprøjten vil være forsynet med randomiseringskode.

10. SIKKERHEDSFORANSTALTNINGER OG PROCEDURER

Det forventes ikke at forsøget medfører nogle særlige risici eller komplikationer for forsøgsdeltagerne. De anvendte lægemiddel (Depo-Medrol) har markedsføringstilladelse i Danmark og bliver ofte brugt som medicinsk behandling af knæartrose. Alle undersøgelser bliver foretaget af relevant uddannet og kvalificeret personale. Der vurderes ikke at være særlige omstændigheder der kan føre til afslutning af forsøget grundet bekymringer for sikkerheden. Derudover bliver forsøget monitoreret af Københavns Universitetshospitals GCP-enhed.

10.1 BIVIRKNINGER OG UTILSIGTEDE HÆNDELSER

Bivirkninger og utilsigtede hændelser er hændelser (fx tegn, symptom, sygdom, syndrom, samtidig sygdom), som opstår hos en forsøgsdeltager efter udsættelse for en intervention, uanset den formodede årsag.

10.2 REGISTRERING AF BIVIRKNINGER OG UTILSIGTEDE HÆNDELSER

Da det anvendte lægemiddel har været anvendt i mere end 50 år (første markedsføringstilladelse 1960 jf. produktresumé) med veldokumenteret effekt og sikkerhed (4;26) antages det at lægemidlets bivirkningsprofil er velbeskrevet. Derfor optages bivirkninger og utilsigtede hændelser som indtræffer efter forsøgets start ikke i CRF.
Kun hændelser der kan kategoriseres som alvorlige utilsigtede hændelser (**SAR** – Serious Adverse Reaction), alvorlige utilsigtede bivirkninger (**SAE** – Serious Adverse Event) eller uventede og alvorlige formodede bivirkninger (**SUSAR** – Suspected Unexpected Serious Adverse Reaction) der indtræffer efter forsøgets start registreres.
I henhold til GCP retningslinjer vil de følgende definitioner blive anvendt:

> **SAR** (serious adverse Reaction – alvorlig bivirkning) eller **SAE** (serious adverse event – alvorlig utilsigtet hændelse): Enhver uønsket helbredsmæssig hændelse eller bivirkning der medfører død, livstruende tilstand, hospitalsindlæggelse, betydelige eller vedvarende invaliditet eller uarbejdsdygtighed eller medfødte anomalier eller misdannelser. Hændelserne vurderes med henblik på deres relation til forsøgsbehandlingen (injektion) som 'relateret' eller 'ikke relateret' jævnfør produktresumeer (Appendiks A, B, C).
>
> **SUSAR** (suspected unexpected serious adverse reaction – uventet og alvorlig formodet bivirkning): En alvorlig bivirkning hvis karakter eller alvorlighed ikke er samstemmende med den relevante produktinformation (Appendiks A, B, C).

Opståen af SUSAR medfører straks-alarmering (dvs. hurtigst muligt og senest 7 dage (ved dødelige eller livstruende SUSAR) og senest 15 dage (ved øvrige SUSAR) efter, at sponsor har fået kendskab hertil) af Sundhedssty-

Downloaded From: http://archinte.jamanetwork.com/ by a University of South Carolina User on 10/26/2015

relsen ved brug af Sundhedsstyrelsens E-blanket til SUSAR indberetning. Desuden rapporteres SUSAR årligt og ved forsøgets afslutning til både Sundhedsstyrelsen og Videnskabsetisk komite.
SAR og SAE rapporteres årligt og ved forsøgets afslutning til både Sundhedsstyrelsen og Videnskabsetisk komite.

10.3 ANTIKONCEPTION

Det kræves, at kvindelige deltagere, som er fertile og seksuelt aktive, benytter sikker antikonception, dette er spiral eller hormonel antikonception (p-piller, implantat, transdermal depotplaster, vaginalring eller depotinjektion). Steril fast partner eller brug af dobbeltbarriere (kondom kombineret med pessar) kan accepteres hvis anvendelsen kan sikres. Forsøgsdeltageren informeres ved informationssamtalen om, at graviditet ved anvendelse af Depo-Medrol generelt frarådes, og at det i forsøgsperioden skal sikres at forsøgsdeltageren ikke bliver gravid.
Sterile eller infertile kvindelige forsøgsdeltagere er fritaget for kravet om brug af antikonception. For at betragtes som steril eller infertil må man almindeligvis være kirurgisk steriliseret (vasektomi/bilateral tubektomi, hysterektomi og bilateral ovarektomi) eller være postmenopausal, defineret som udebleven menstruation i mindst 12 måneder før studie-indrullering.
Hvorvidt forsøgsdeltageren er fertil og hvorvidt og hvorvidt patienten er seksuelt aktiv afklares ved en lægelig vurdering baseret på samtale med forsøgsdeltageren ved screeningsundersøgelsen. Ydermere indskærpes det overfor forsøgsdeltageren, at hvis status for fertilitet og seksuel aktivitet ændrer sig fra ikke til stede til indtrådt i en forsøgsperioden skal forsøgsdeltageren underrette den ansvarlige læge herom, således at der løbende tages stilling til behov for antikonception.

11 VIDENSKABSETISK REDEGØRELSE

11.1 GENERELLE OVERVEJELSER

Før screening informeres alle potentielle forsøgsdeltagere både mundtligt og skriftligt om projektets formål, forløb samt eventuelle risici, ulemper og fordele ved deltagelse. Desuden uddeles folderne 'Før du beslutter dig' og 'Dine rettigheder som forsøgsperson i et biomedicinsk forskningsprojekt'. Alle deltagerne informeres om deres rettigheder til at kunne trække sig ud af studiet til enhver tid uden at dette må få konsekvenser for deres videre eller fremtidige undersøgelser og/eller behandling på Frederiksberg Hospital eller af nogle af medlemmerne i studiegruppen. Efter informationen er uddelt, læst og forstået, afgives informeret samtykke frivilligt af deltageren ved at underskrive en samtykkeerklæring, før deltagelse kan finde sted.

11.2 PROCEDURER FOR AFGIVELSE AF DEN MUNDTLIGE INFORMATION

Ved henvendelse træffes en aftale om tid og sted for afholdelse af informationssamtalen. Det skal i denne sammenhæng understreges at der er tale om en forespørgsel om deltagelse i et videnskabeligt forsøg, samt at den potentielle forsøgsdeltager har ret til at medbringe en bisidder til informationssamtalen. Det skriftlige informationsmateriale fremsendes per e-mail eller post så den potentielle forsøgsdeltager har mindst 24 timer til gennemlæsning af materialet inden informationssamtalen.
Den mundtlige information tager udgangspunkt i den skriftlige information og afgives i et let forståeligt sprog uden tekniske eller værdiladede udtryk. Informationen afgives på en hensynsfuld måde, der er tilpasset den enkelte potentielle forsøgsdeltager. Det tilstræbes at samtalen foregår uden forstyrrelser. Det er forsøgslederens ansvar at sikre, at den potentielle forsøgsdeltager har forstået informationen. Ved informationssamtalen deltager forsøgslederen og i dennes fravær kan projektkoordinatoren eller den klinisk ansvarlige deltage.

Downloaded From: http://archinte.jamanetwork.com/ by a University of South Carolina User on 10/26/2015

11.3 SKRIFTLIG INFORMATION

Der er udarbejdet et skriftligt informationsmateriale der er vedlagt denne protokol som Appendiks E.

11.4 INFORMERET SAMTYKKE

Samtykke til forsøgsdeltagelse afgives på baggrund af den skriftlige og mundtlige information.
Der er udarbejdet en samtykkeerklæring til underskrift (Appendiks F). Samtykkeerklæringen skal underskrives og dateres af forsøgsdeltagerne forud for deltagelse i undersøgelsen. En kopi af samtykkeerklæringen udleveres til forsøgsdeltageren. Forsøgslederen, studiekoordinatoren og den klinisk ansvarlige kan modtage samtykket. Inden et informeret samtykke modtages, skal det sikres at de potentielle forsøgsdeltagere har haft betænkningstid til at overveje deres deltagelse.

11.5 FULDMAGT TIL JOURNALGENNEMGANG

Fuldmagt til at Sundhedsstyrelsen kan få adgang til patientjournaler med henblik på inspektion og kontrol af forsøget indhentes inden inklusion i forsøget (Appendiks G).

11.6 VIDENSKABSETISK REDEGØRELSE FOR DE PLANLAGTE INTERVENTIONER

11.6.1 Intraartikulær corticosteroid og saltvand

Intraartikulær corticosteroid er en anerkendt og veletableret behandling af knæ OA. Behandlingen medfører reduktion i den smertegivende inflammation og er således gavnlig for patienten. Saltvandsinjektioner har også vist sig at have en vis klinisk effekt hvorfor den blindede gruppetildeling ikke synes at være forbundet med videnskabsetiske problemstillinger. Da alle patienter screenes for kontraindikationer mod corticosteroid behandling anses injektionsinterventionerne ikke at være forbundet med videnskabsetiske problemstillinger. Som ved alle andre injektionsprocedurer er der risiko for efterfølgende infektion. Alle injektioner udføres efter Frederiksberg Hospitals hygiejneforeskrifter, og infektionsrisikoen er ekstrem lille (sjældnere end én gang pr. 10.000 injektioner).

11.6.2 Fysioterapeutisk træning

Den gavnlige effekt af træning på smerter ved knæ OA er veldokumenteret, og træning anbefales som primær behandling af knæ OA. Desuden er træning påvist at have store gavnlige effekter for den enkeltes sundhed, hvilket anses som værende en fordel for forsøgsdeltagerne. Der kan dog være risici forbundet med træning, men da alle deltagere modtager træning, denne udføres superviseret af uddannet personale (fysioterapeuter), og forsøgsdeltagerne screenes for eventuelle forhold der kontraindicerer træning, anses den planlagte træningsintervention ikke at være forbundet med videnskabsetiske problemstillinger.

11.7 VIDENSKABSETISK REDEGØRELSE FOR DE PLANLAGTE MÅLEMETODER

11.7.1 Patientrapporterede effektmål (spørgeskemaer)

Undersøgelserne er non-invasive, og er ikke forbundet med nogen former for risici. Metoden anses for at være uden etiske problemstillinger.

11.7.2 Evaluering af perifere og centrale smertemekanismer

Undersøgelserne er non-invasive, og er ikke forbundet med nogen former for risici. Undersøgelserne er i sagens natur smertefulde, men metoden anses for at være uden etiske problemstillinger.

11.7.3 Evaluering af proinflammatoriske cytokiner i ledvæske, blod og urin

Aspiration af ledvæske indebærer indførelse af en kanyle i knæleddet. Dette kan være forbundet med et ube-

Downloaded From: http://archinte.jamanetwork.com/ by a University of South Carolina User on 10/26/2015

hag i forbindelse med indstikket. Proceduren udføres ultralydsvejledt af en speciallæge i reumatologi eller radiologi og følger Frederiksberg hospitals gængse hygiejneforeskrifter for aseptisk injektionsteknik. Ved punktering af huden er der dog altid risiko for efterfølgende infektioner, men proceduren er en standardbehandling og incidensen af infektioner er meget lille. Metoden anses for sikker og uden etiske problemstillinger.
Der skønnes ikke at vare risiko forbundet med udtagning af blodprøve eller aflevering af urinprøve. Blodprøven består i 22,5 ml per gang eller i alt 45 ml.
Både brusknedbrydningsstofferne og inflammationsstofferne bestemmes ved enzymmålemetode (ELISA) i eget laboratorium ved anvendelse af et målekit. Der skønnes ikke at være videnskabsetiske problemstillinger forbundet med metoden.

11.7.4 Evaluering af billeddiagnostiske (MR) tegn på inflammation
Bortset fra indgivelse af kontraststof er MR-scanning non-invasiv og er ikke forbundet med nogen former for risici. Det anvendte kontraststof (Prohance) er kategoriseret som et lav-risikostof og forsøgsdeltagerne undersøges ved blodprøve (s-creatinin og e-GFR) for kontraindikationer imod anvendelse af kontraststof. Ved kontraindikation gennemføres MR-scanningen ikke men dette udelukker ikke forsøgsdeltageren for øvrig deltagelse i forsøget. Ved anvendelse af kontraststoffet kan der forekomme bivirkninger, hvoraf den hyppigst forekommende bivirkning er kvalme. Den intravenøse injektion skønnes ikke at være forbundet med risiko. Personer med klaustrofobi eller tendens dertil kan opleve ubehag ved lejring i scanneren. Metoden anses for at være uden etiske problemstillinger.

11.7.5 Muskelstyrke, fysisk funktionsniveau, funktionel smertetest, ultralydsundersøgelse og biomekanisk ganganalyse
Undersøgelserne er non-invasive, og er ikke forbundet med nogen former for risici. Metoderne anses for at være uden etiske problemstillinger.

11.8 VIDENSKABSETISK GODKENDELSE
Denne protokol, samtykkeerklæringen, skriftlig patientinformation, annoncemateriale og anden relevant information indsendes af forsøgslederen til godkendelse ved den lokale videnskabsetiske komite. Dette studie udføres i overensstemmelse med gældende dansk lovgivning, Helsinki deklarationen, og forskrifter udstukket af videnskabsetisk komite.
Forsøgslederen er ansvarlig for at informere videnskabsetisk komite om relevante protokolændringer og tillæg, samt at afrapportere om projektets progres.

12 OVERHOLDELSE AF LOVE OG REGLER
Forsøget udføres i overensstemmelse med nærværende protokol samt gældende myndighedskrav og relevant lovgivning på området.

13. PROJEKTETS AFVIKLING
13.1 ADMINISTRATIV STRUKTUR
Parker Instituttet er ansvarlig for udførelsen af dette projekt. Sponsor bærer det overordnede ansvar for projektets gennemførsel og publicering af resultaterne. Investigator sikrer at forsøgsdeltagernes sikkerhed ikke kompromitteres.

13.2 BEHANDLING AF FORTROLIGE OPLYSNINGER
Alle oplysninger vedrørende forsøgspersonernes identitet beskyttes efter lov om behandling af personoplys-

Downloaded From: http://archinte.jamanetwork.com/ by a University of South Carolina User on 10/26/2015

ninger og sundhedsloven. Så snart et anonymiseret projekt-id nummer er etableret og alle personrelaterede data er anonymiserede destrueres personfølsomme oplysninger.

13.3 ANMELDELSE TIL DATATILSYNET

Eftersom projektet udføres på en hospitalsafdeling, betragtes projektet som "offentligt", jf. Datatilsynets vejledning. Derfor varetages anmeldelsen af projektet til Datatilsynet af den offentlige myndighed som afdelingen hører under, i nærværende tilfælde Region Hovedstaden. Således foretages anmeldelse af nærværende projekt til "Enheden for Informationssikkerhed" under Region Hovedstadens IT afdeling, der varetager den videre anmeldelse til Datatilsynet.

13.4 ANMELDELSE TIL LÆGEMIDDELSTYRELSEN

Eftersom projektet omfatter medicinsk behandling af knæ OA anmeldes projektet til Lægemiddelstyrelsen.

13.5 ØKONOMISKE FORHOLD

Projektet er initieret af sponsor, fysioterapeut ph.d. Marius Henriksen, samt af dennes overordnede på Parker Instituttet professor, dr.med. Henning Bliddal der også er investigator på projektet.
Projektet er støttet økonomisk af

- Statens Forskningsråd for Sundhed og Sygdom (1.8 mio. kroner til løn af projektmedarbejdere)
- The Oak Foundation (Parker Instituttets basisbevilling hvoraf et ikke nærmere specificeret beløb er afsat)
- Danske Fysioterapeuter (440.000 kroner til løn af projektmedarbejdere og driftsomkostninger)
- Lundbeckfonden (200.000 kroner til løn af projektmedarbejdere og driftsomkostninger)

, hvilket vil fremgå af det skriftlige informationsmateriale.
Hverken den forsøgsansvarlige, andre ansatte på dennes institution, eller øvrige medarbejdere tilknyttet projektet har økonomiske interesser i projektets resultater.
Finansiering af projektet er en fortløbende proces. Eventuelle fremtidige bevillinger/sponsorater vil blive oplyst forsøgsdeltagerne ligesom informationer herom vil blive indskrevet i det skriftlige informationsmateriale.

13.6 INFORMATION TIL FORSØGSDELTAGERE

Inkluderede og potentielle forsøgsdeltagere kan indhente yderligere information om forsøget ved henvendelse til sponsor, studiekoordinatoren eller investigator pr. telefon eller e-mail. Den klinisk ansvarlige kan også kontaktes i forbindelse med spørgsmål af medicinsk faglig karakter der er relateret til deltagelse i projektet. Den skriftlige deltagerinformation, forsøgsprotokollen og lægmandsprotokollen vil blive udleveret ved henvendelse til sponsor, den forsøgsansvarlige eller studiekoordinatoren.

13.7 PUBLIKATION AF RESULTATER

I henhold til dansk lov offentliggøres resultaterne i form af videnskabelige artikler i internationale videnskabelige tidsskrifter med peer-review. Publikation af resultaterne vil foregå fortløbende efterhånden som projektet afsluttes og data analyseres. Såvel positive som negative resultater vil blive offentliggjort. Ved tvivl om forfatterskab og –rækkefølge afgøres denne med Parker Instituttets bestyrelsesformand som opmand.

13.8 KVALITETSKONTROL OG –SIKRING

Nærværende forsøg udføres i overensstemmelse med ICH-GCP. Københavns Universitetshospitals GCP-enhed monitorerer forsøget. En eller flere autoriserede repræsentanter fra GCP-enheden vil periodisk besøge studiesitet for at inspicere forsøgsdata, patientjournaler og CRF.

Downloaded From: http://archinte.jamanetwork.com/ by a University of South Carolina User on 10/26/2015

Investigator bemyndiger GCP-enhedens og Sundhedstyrelsens repræsentanter til at inspicere forsøgsfaciliteter og for forsøget relevante journaler og dataoptegnelser.

13.9 FORSØGSDELTAGERFORSIKRING

Forsøgsdeltagerne i forsøget er dækket at Patientforsikringen.

13.10 TIDSPLAN

Juni - August 2012

- Datatilsynet, Lægemiddelstyrelsen og de Videnskabsetiske Komiteer for Region Hovedstaden ansøges.
- Instruktion og træning af forsøgspersonale.

Oktober 2012 - Marts 2013

- Rekruttering af forsøgsdeltagere

December 2013

- Sidste forsøgsdeltager afsluttes i forsøget

Januar 2014 – Marts 2014

- Dataanalyse

14. OVERSIGT OVER APPENDICES

Appendiks A: Lægemiddelstyrelsens Produktresumé for Depo-Medrol®
Appendiks B: Lægemiddelstyrelsens Produktresumé for Lidokain
Appendiks C: Lægemiddelstyrelsens Produktresumé for Natriumklorid Isotonisk
Appendiks D: Skematisk oversigt over planlagte målinger
Appendiks C: Skriftligt informationsmateriale
Appendiks F: Samtykkeerklæring

Downloaded From: http://archinte.jamanetwork.com/ by a University of South Carolina User on 10/26/2015

15. REFERENCER

(1) Ostergaard M, Halberg P. [Intra-articular glucocorticoid injections in joint diseases]. Ugeskr Laeger 1999 Feb 1;161(5):582-6.
(2) Arroll B, Goodyear-Smith F. Corticosteroid injections for osteoarthritis of the knee: meta-analysis. BMJ 2004 Apr 10;328(7444):869.
(3) HOLLANDER JL, BROWN EM, Jr., JESSAR RA, BROWN CY. Hydrocortisone and cortisone injected into arthritic joints; comparative effects of and use of hydrocortisone as a local antiarthritic agent. J Am Med Assoc 1951 Dec 22;147(17):1629-35.
(4) Ostergaard M, Halberg P. Intra-articular corticosteroids in arthritic disease: a guide to treatment. BioDrugs 1998 Feb;9(2):95-103.
(5) Zhang W, Nuki G, Moskowitz RW, Abramson S, Altman RD, Arden NK, et al. OARSI recommendations for the management of hip and knee osteoarthritis: part III: Changes in evidence following systematic cumulative update of research published through January 2009. Osteoarthritis Cartilage 2010 Apr;18(4):476-99.
(6) Sharma L, Kapoor D, Issa S. Epidemiology of osteoarthritis: an update. Curr Opin Rheumatol 2006 Mar;18(2):147-56.
(7) HOLLANDER JL, BROWN EM, Jr., JESSAR RA, BROWN CY. Hydrocortisone and cortisone injected into arthritic joints; comparative effects of and use of hydrocortisone as a local antiarthritic agent. J Am Med Assoc 1951 Dec 22;147(17):1629-35.
(8) Jordan KM, Arden NK, Doherty M, Bannwarth B, Bijlsma JW, Dieppe P, et al. EULAR Recommendations 2003: an evidence based approach to the management of knee osteoarthritis: Report of a Task Force of the Standing Committee for International Clinical Studies Including Therapeutic Trials (ESCISIT). Ann Rheum Dis 2003 Dec;62(12):1145-55.
(9) Petersen AM, Pedersen BK. The anti-inflammatory effect of exercise. J Appl Physiol 2005 Apr;98(4):1154-62.
(10) Helmark IC, Mikkelsen UR, Borglum J, Rothe A, Petersen MC, Andersen O, et al. Exercise increases interleukin-10 levels both intraarticularly and peri-synovially in patients with knee osteoarthritis: a randomized controlled trial. Arthritis Res Ther 2010 Jul 1;12(4):R126.
(11) van Baar ME, Dekker J, Oostendorp RA, Bijl D, Voorn TB, Bijlsma JW. Effectiveness of exercise in patients with osteoarthritis of hip or knee: nine months' follow up. Ann Rheum Dis 2001 Dec;60(12):1123-30.
(12) Roos EM, Toksvig-Larsen S. Knee injury and Osteoarthritis Outcome Score (KOOS) - validation and comparison to the WOMAC in total knee replacement. Health Qual Life Outcomes 2003;1:17.
(13) Imamura M, Imamura ST, Kaziyama HH, Targino RA, Hsing WT, de Souza LP, et al. Impact of nervous system hyperalgesia on pain, disability, and quality of life in patients with knee osteoarthritis: a controlled analysis. Arthritis Rheum 2008 Oct 15;59(10):1424-31.
(14) Arendt-Nielsen L, Nie H, Laursen MB, Laursen BS, Madeleine P, Simonsen OH, et al. Sensitization in patients with painful knee osteoarthritis. Pain 2010 Jun;149(3):573-81.
(15) Graven-Nielsen T, Arendt-Nielsen L. Assessment of mechanisms in localized and widespread musculoskeletal pain. Nat Rev Rheumatol 2010 Jul 27.
(16) Waehrens E, Bliddal H, nneskiold-Samsoe B, Lund H, Fisher A. Differences between questionnaire- and interview-based measures of activities of daily living (ADL) ability and their association with observed ADL ability in women with rheumatoid arthritis, knee osteoarthritis, and fibromyalgia. Scand J Rheumatol 2012 Jan 30.
(17) Hochman JR, Gagliese L, Davis AM, Hawker GA. Neuropathic pain symptoms in a community knee OA cohort. Osteoarthritis Cartilage 2011 Jun;19(6):647-54.

Downloaded From: http://archinte.jamanetwork.com/ by a University of South Carolina User on 10/26/2015

(18) Nie H, rendt-Nielsen L, Andersen H, Graven-Nielsen T. Temporal summation of pain evoked by mechanical stimulation in deep and superficial tissue. J Pain 2005 Jun;6(6):348-55.
(19) Polianskis R, Graven-Nielsen T, rendt-Nielsen L. Spatial and temporal aspects of deep tissue pain assessed by cuff algometry. Pain 2002 Nov;100(1-2):19-26.
(20) Hunter DJ, Lo GH, Gale D, Grainger AJ, Guermazi A, Conaghan PG. The reliability of a new scoring system for knee osteoarthritis MRI and the validity of bone marrow lesion assessment: BLOKS (Boston Leeds Osteoarthritis Knee Score). Ann Rheum Dis 2008 Feb;67(2):206-11.
(21) Boesen M, Kubassova O, Cimmino MA, Østergaard M, Taylor PC, Danneskiold-Samsoe B, et al. Dynamic Contrast Enhanced MRI Can Monitor the Very Early Inflammatory Treatment Response upon Intra-Articular Steroid Injection in the Knee Joint: A Case Report with Review of the Literature. Arthritis 2011 Feb 1;2011:1-8.
(22) Kubassova O, Boesen M, Cimmino MA, Bliddal H. A computer-aided detection system for rheumatoid arthritis MRI data interpretation and quantification of synovial activity
3. Eur J Radiol 2009 May 1.
(23) Vaughan CL, Davis BL, O'Connor JC. Dynamics of Human Gait. 2nd ed. Cape Town: Kiboho Publishers; 1999.
(24) Nigg BM. Biomechanics of the musculo-skeletal System. Chichester, England: John Wiley & Sons Ltd; 1994.
(25) Enright PL, McBurnie MA, Bittner V, Tracy RP, McNamara R, Arnold A, et al. The 6-min walk test: a quick measure of functional status in elderly adults. Chest 2003 Feb;123(2):387-98.
(26) HOLLANDER JL, BROWN EM, Jr., JESSAR RA, BROWN CY. Hydrocortisone and cortisone injected into arthritic joints; comparative effects of and use of hydrocortisone as a local antiarthritic agent. J Am Med Assoc 1951 Dec 22;147(17):1629-35.

Downloaded From: http://archinte.jamanetwork.com/ by a University of South Carolina User on 10/26/2015
