# Supplementary material for: Intra-Articular Corticosteroids in Addition to Exercise for Reducing Pain Sensitivity in Knee Osteoarthritis: Exploratory Outcome from a Randomized Controlled Trial
Source: PLoS One. 2016 Feb 12;11(2):e0149168. doi: 10.1371/journal.pone.0149168 (PMC4752217; doi:10.1371/journal.pone.0149168)
Supplement: S2 Protocol — (DOC) [file pone.0149168.s004.doc]

RETROSPECTIVELY TRANSLATED VERSION OF PRINCIPIAL METHODOLOGICAL SECTIONS OF TRIAL PROTOCOL FOR THE TRIAL

**COMBINED INTRA ARTICULAR CORTICOSTEROID AND PHYSIOTHERAPEUTIC EXERCISE IN PATIENTS WITH OSTEOARTHRITIS OF THE KNEE: A RANDOMISED CLINICAL TRIAL**

Please note that this version of the protocol is not a translation of the complete trial protocol but only reflects principal methodological sections of the protocol. Therefore this document does not provide a complete and detailed picture of the methodology of the trial.

The translation was made by the authors (i.e. not a professional translation) upon editorial request from the journal PLOS ONE. We (the authors) first declined to translate the protocol retrospectively as we believe it is disingenuous and wasteful to translate protocols retrospectively – especially by us, the protocol authors. However, the journal requested key methodological issues to be translated to ease the assessment of our submission. As a consequence, we followed the request.

The original and complete protocol is in Danish, has been submitted together with the manuscript, and is also available from the principal investigators.

The primary and main secondary outcomes of the trial have been published in full in *JAMA Internal Medicine 2015;175(6):923-930.*

**Principal investigators**

Marius Henriksen, physiotherapist, Msc, PhD
The Parker Institute
Copenhagen University Hospital at Frederiksberg and Bispebjerg
Ndr. Fasanvej 57
DK-2000 Copenhagen F
Denmark
Telephone: +45 3816 4160
Telefax: +45 3816 4159
Email: [marius.henriksen@regionh.dk](mailto:marius.henriksen@regionh.dk)

Henning Bliddal, professor, MD, DMSc
The Parker Institute
Copenhagen University Hospital at Frederiksberg and Bispebjerg
Ndr. Fasanvej 57
DK-2000 Copenhagen F
Denmark
Telephone: +45 3816 4151
Telefax: +45 3816 4159
Email: [henning.bliddal@regionh.dk](mailto:henning.bliddal@regionh.dk)

# BACKGROUND

Osteoarthritis (OA) of the knee is a very common chronic joint disorder (1) associated with risk of mobility disability greater than that due to any other medical condition in people ages ≥65. Knee OA is associated with increasing age, and thus the societal burden is expected to increase with the increasing life time expectancy of the population, thus posing a major socioeconomic and healthcare burden. As no cure exists, management aims to reduce pain, improve function, and enhance quality of life. The recommended hierarchy of management should consist of non-pharmacological treatments in first line, then the add-on of drugs, and then, if necessary, surgery (2).

Non-pharmacological treatments include a range of modalities, including exercise therapy. Systematic reviews and meta-analyses consistently show beneficial effects of exercise therapy on pain and physical function in patients with knee OA (3;4). Accordingly clinical guidelines state that therapeutic exercise is central in the management of patients with knee OA. The 3^rd^ EULAR (European League Against Rheumatism) recommendation acknowledges the crucial importance of exercise therapy (5), and the international OA society (OsteoArthritis Research Society International; OARSI) recommends exercise therapy as an essential treatment for knee OA (4). The effect size of exercise therapy is of the same magnitude as most pharmacological treatments but is often without adverse effects (4). Although the analgesic mechanism behind exercise therapy is largely unknown, experimental studies suggest potential anti-inflammatory effects (6;7).

Pharmacological treatments mainly focus on pain relief. Systemic treatment by paracetamol or non-steroidal anti-inflammatory drugs (NSAIDs) is common and readily available to the patients. NSAIDs should be administered under careful control and attention to adverse effects, especially cardiovascular, renal, and gastrointestinal. Local pharmacological treatment of the afflicted knee joint is recommended by means of intra-articular injections of corticosteroids, which is considered as a standard medical treatment of knee OA and is included in established guidelines for management of knee OA (4). Injection of corticosteroids has been known for more than 50 years with well documented effects and safety (8;9). A meta analysis of randomised placebo controlled trials of high methodological quality (10) showed a pooled relative risk of symptomatic improvement 16-24 weeks after intra-articular corticosteroid injection of 2.09 (95% confidence interval 1.2 to 3.7) and a number-needed-to-treat of 4.4. The pooled relative risk of symptomatic improvement for up to 2 weeks after injection was 1.66 (95% confidence interval 1.37 to 2.0) with a number-needed-to-treat between 1.3 and 3.5 in the trials showing statistically significant results. The evidence support short term (up to 2 weeks) and long term (16-24 weeks) symptomatic improvement by intra-articular corticosteroid injection in patients with knee OA (10) .

Non-surgical management of patients with knee OA should take an individual approach and will typically consist of combinations of non-pharmacological and pharmacological treatments. While the two treatment approaches have been investigated separately in numerous clinical trials, the efficacy of a combined pharmacological and non-pharmacological approach is not known, despite the high rank of such combined treatment approach on the recommended hierarchy of management (2). From a physiotherapy practice point of view, it is not uncommon to receive patients for exercise therapy that have had an intra-articular injection of corticosteroid at their general practitioner or practicing rheumatologist. This encumbers clinical goal setting, valid assessment of exercise treatment response, rational exercise adjustments, and comparison with scientific literature in the field. Thus, the current non-surgical palliation approach seem multidisciplinary, yet uncoordinated, and with no evidence in support.

Thus, the purpose of this study is to assess the effects of exercise therapy in combination with intra-articular corticosteroid injections on patient reported pain and function in patients with knee OA. We hypothesize that the combined treatment approach (exercise therapy preceded by intra-articular injection of corticosteroid) enhances the clinical outcome compared to exercise therapy preceded by a placebo injection.

# STUDY DESIGN

This is a randomised, parallel-group, double-blind study assessing the combined effects of intra-articular corticosteroid injection and exercise therapy on patient reported pain in patients with knee OA. The participants will be randomised (1:1) to 1 active group and 1 control group:

- Intra-articular corticosteroid injection followed by exercise therapy supervised by trained physiotherapists
- Intra-articular placebo injection (sterile isotonic saline) followed by exercise therapy supervised by trained physiotherapists

The participants are assessed at baseline (before randomisation), after 14 weeks (2 weeks to allow for the corticosteroid to have its effect + 12 weeks of exercise therapy), and again after 26 weeks (12 weeks after termination of exercise). The exercise is commenced 2 weeks after the injection.

## RATIONALE FOR STUDY DESIGN

The study is designed to assess the effect of combined intra-articular corticosteroid treatment to exercise therapy on patient reported pain in patients with knee OA. To assess the combined therapy a control group is included in the study design that receives a placebo injection (saline) followed by exercise therapy.

The exercise therapy consists of facility based exercise 3 times per week for 12 weeks following standard recommendations for therapeutic exercise to patients with knee OA.

We choose to commence the exercise therapy 2 weeks after the injection because our clinical experience suggests that maximum clinical effect of intra-articular corticosteroid is reached after approximately 2 weeks. The estimated effect duration of intra-articular corticosteroid therapy is between 8-56 days (11), whereas the effect duration of a combined treatment is unknown. It is known that the beneficial clinical effects of exercise therapy gradually decrease after exercise termination (12). To assess if the combination therapy prolongs the beneficial effects, the participants are assessed 12 weeks after exercise termination (26 weeks after baseline assessment).

# OUTCOMES

### Primary outcome

- Change from baseline to week 14 in patient reported pain assessed by pain subdomain in the Knee Injury and Osteoarthritis Outcome Score (KOOS) questionnaire (13)

### Secondary outcomes

- Change from baseline to week 2 and 26 in patient reported pain assessed by the KOOS questionnaire sub domain pain
- Change from baseline to week 2, 14, and 26 in patient reported symptoms, function, quality of life and, function during sports and recreation assessed by the KOOS questionnaire
- Change from baseline to week 2, 14, and 26 in patient reported function during activities of daily living (ADL-Q questionnaire)
- Change from baseline to week 2, 14, and 26 in indications of presence of neuroapthic pain (PainDetect questionnaire)
- Change from baseline to week 14, and 26 in a functional pain test
- Change from baseline to week 14 and 26 in pain sensitivity measured by cuff pressure algometry
- Change from baseline to week 14, and 26 in quadriceps and hamstring muscle strength
- Change from baseline to week 14, and 26 in 6-minute walk distance
- Change from baseline to week 14, and 26 in inflammation markers in blood, urine and joint fluid
- Change from baseline to week 14, and 26 in inflammation assessed by delayed contrast enhanced magnetic resonance imaging (DCE-MRI)

**Exploratory outcomes**

- Change from baseline to week 14, and 26 in muscle strength
- Change from baseline to week 14, and 26 in 6 minute walk test

**DESCRIPTION OF THE OUTCOMES**

Knee Injury and Osteoarthritis Outcome Score (KOOS)

The patients’ own perception of pain, symptoms, function in daily activities, quality of life, and function during sports and recreational activities are assessed using the questionnaire ’Knee Injury and Osteoarthritis Outcome Score’ (KOOS) (12). A Danish version of the questionnaire is used.

ADL-Q questionnaire

The patients’ own perception of functional limitations in activities of daily living is assessed using the ADL-Q questionnaire (16). Der anvendes en dansk udgave af spørgeskemaet, der er valideret overfor det originale. A Danish version of the questionnaire is used.

PainDetect questionnaire

Indications of presence of neuroapthic pain are assessed by the ’PainDetect’ questionnaire (17). A Danish version of the questionnaire is used.

Functional pain test

The patients’ knee pain intensity during standardized physical test is recorded on a 0-10 numerical rating scale. The tests encompass walking, sit-to-stand, stair walking.

Pain sensitivity (Computer controlled cuff pressure algometry)

Pressure-pain sensitivity is estimated by computerized cuff pressure algometry, which consists of a double-chamber tourniquet cuff (VBM Medizin-technik GmbH) and a computer-controlled air compressor (DoloCuff, Unique Electronic Aps). The cuff is wrapped around the calf at the bulky part of the gastrocnemius muscle. Both chambers of the cuff are inflated at 1 kPa/second until the patient reports that the sensation of pressure starts to become painful. The pressure at this point defines the pressure-pain threshold (PPT; measured in kPa). The PPT is registered 3 times separated by at least 60 seconds, and the average used for analysis. Temporal summation (TS) of pressure–pain is assessed on the same site as the PPT using constant pressure stimulation at 125% of the PPT during 6 minutes. Participants continuously rate their pain intensity during the test on an electronic 0–100-mm visual analog scale (VAS), with 0 and 100 mm anchored as “no pain” and “worst pain imaginable”, respectively. The VAS signal is sampled by a computer at 10-Hz. TS of pressure–pain is quantified as the area under the time-VAS curve.

Inflammation markers in blodd, urine and joint fluids

Under ultrasound guidance excessive joint fluid is aspirated at each assessment. Further, participants will have fasting blood samples taken and deliver urine samples

Inflammation markers assessed by ultrasound imaging

The ultrasound imaging includes soft tissue, menisci, and synovial hypertrophy and perfusion. The examinations are done on a General Electric Logiq9™ ultrasound scanner.

Inflammation markers assessed by Magnetic Resonance (MR) imaging

An MR protocol used to provide images of cartilage, bone and surrounding structures is applied. A Siemens 3Tesla Verio scanner is used. Scans are done before, during, and after intravenous injection of contrast agent (single dose Gadolinium, 0.05mmol, Prohance, Bracco International, Holland). Details about the MR protocol and analyses are in a separate document.

Biomechanical gait analysis

The participants’ gait patterns are assessed in a gait laboratory according to international standards.

Muscle strength

Maximal muscle strength of the quadriceps and hamstrings are measured using an isokinetic dynamometer. Muscle strength is measured isometrically (0 degress/sec) at 60 degrees of knee flexion. Three measurements are done.

Six-minute walk test

The six-minute walk test is used as a reflection of the overall level of physical functioning. The test assesses both cardiovascular and musculoskeletal function as a whole. The participant walks a 100 meter indoor track as many times as possible in 6 minutes. The distance covered is the test results.

# PARTICIPANTS

## Recruitment

Participants are recruited from the osteoarthritis outpatient clinic at Copenhagen University Hospital at Bispebjerg and Frederiksberg, Denmark, and from advertisements in local newspapers, bulletin boards, webpages etc.

## INCLUSION CRITERIA

Potential participants must fulfil the following inclusion criteria in order to be eligible for participation:

- Age above 40 years
- Diagnosed with knee OA according to diagnostic criteria
- Radiologically verified changes in accordance with tibiofemoral osteoarthritis
- Pain of at least 40 mm on a 0-100 mm VAS when asked “how painful is your knee during level walking?”
- Clinical signs of localised inflammation of the knee
- Willing to suspend current pain medication during assessments in the study
- Willing to participate in all assessments
- Willing to participate in the exercise program
- 20≤ Body Mass Index (BMI) ≤35 kg/m^2^
- Willing to adhere to the rules about concurrent medication and treatment
- Speak, read and write Danish

## EXCLUSION CRITERIA

Individuals who fulfil any of the following exclusion criteria are not eligible for participation:

- Participation in physiotherapeutic exercise for knee OA within 3 months
- Treatment with intra-articular corticosteroid in knee within 3 months
- Exercise counter indicated
- intra-articular corticosteroid counter indicated
- Pregnant or breast feeding
- Current or previous symptoms of autoimmune disease
- Planned surgery during study period
- Current or previous diagnosis, signs, or symptoms of significant cardiovascular disease, including, but not restricted to
- Ischaemic heart disease
- Arteriosclerosis
- Medical conditions that precludes exercise, including but not restricted to
- Chronic or congenital heart disease
- Severe Asthma
- Chronic Obstructive Pulmonary Disease
- Previous or current diagnosis, sign, or symptoms of significant neurological disease including but not restricted to:
- Cerebral embolism
- Cerebral aneurism
- Clinically significant head trauma within one year
- Peripheral neuropathy
- Epilepsy or seizures
- Severely impaired balance
- Alcohol or drug abuse within 5 years
- Previous or current diagnosis, sign or symptoms of significant psychiatric disorder
- Pain syndromes, e.g. fibromyalgia
- Regional pain caused by lumbar or cervical nerve root compression with radiculopathy or at risk of developing such
- Any other condition that by the opinion of the investigator makes a potential participant ineligible for participation, e.g. due to excess risk

# INTERVENTIONS

## INJECTIONS

Intra-articular corticosteroid (COR):
Injections are made under ultrasound guidance. Before injection any excess joint fluid will be aspirated. 1 ml methylprednisolone corticosteroid (40 mg Depo-Medrol®) mixed with 4 ml Lidocaine (10 mg/ml, SAD) is injected.

Intra-articular saline (SAL):
Injections are made under ultrasound guidance. Before injection any excess joint fluid will be aspirated. 1 ml isotonic saline mixed with 4 ml Lidocaine (10 mg/ml, SAD) is injected.

To ensure blinding of both participant and the person giving the injection the syringes are prepared by an unblended study nurse, according to the randomization (COR or SAL) in the absence of blinded personnel and participants. Because the corticosteroid mixture is milky white and the saline mixture is transparent the syringes are wrapped with opaque tape to hide the content. After this the syringe is handed to the blinded person that does the injection. The syringes will be marked with randomization codes.

## EXERCISE

The exercise program includes muscle strengthening and coordination exercises. Before exercising a 10 minutes warm-up is done on an ergometer bike at moderate intensity. The exercises are facility based and supervised by a trained physiotherapist. Details of the program exist in a separate document.

# RANDOMISATION, BLINDING, AND MINIMISING BIAS

### Group allocation and randomisation

The Parker Institute’s statistician is responsible for development of a randomization list before commencement of the study. The randomization will be done in blocks of 2.6 in order to account for any seasonal variations. The randomization is equal (1:1), with 50 participants in each group. A duplicate of the randomization list is kept by the administration at The Parker Institute.

Upon inclusion of a participant, signed informed consent and complete baseline assessments, the unblended study nurse will give the participant the next available randomization number from the randomization list.

The participant is randomised to either the COR or the SAL group using envelope based randomization. Before the study start a folder containing 100 envelopes is created. Each envelope contains a piece of paper with either “COR” or “SAL” written on it. The order of the envelope corresponds to the randomization list. It is ensured that the envelopes are opaque and sealed. The envelopes are numbered consecutively from 1-100 and placed in the folder with no. 1 in front ant no. 100 in the back. The number on the envelope corresponds to the randomization number. The folder is kept at the unblended study nurse’s office. A duplicate set of envelopes is kept with the administration at The Parker Institute.

When a participant has completed all baseline assessments , the unblinded study nurse will take an envelope from the folder, starting from the front. The participant’s name, date of birth, screening number, and date of inclusion is written on the envelope.

After randomisation the syringes are prepared.

# STATISTICAL ANALYSES

All analyses are done on the intention-to-treat (ITT) population. All statistical tests are 2-tailed with alpha = 0.05.
Continuous outcomes
Changes from baseline are compared between groups. Repeated measures analysis of covariance with adjustment for the baseline value will be used. Exploratory analyses with selected demographical and baseline variables as covariates may be done to assess the effects of other factors on the outcomes.

Missing data
Missing data were replaced using multiple imputation (5 iterations) using age, gender, BMI, baseline scores, and group allocations (masked) as predictors.

# SAMPLE SIZE

This study is designed to compare the participants allocated to corticosteroid and those allocated to placebo. Assuming that corticosteroid would produce a reduction in KOOS pain that was 10 points larger than the placebo, with a standard deviation of 15 points, we calculate 100 patients in the intention-to-treat (ITT) population is needed to test a two tailed hypothesis with 91% power at a 5% statistical significance level.

# RERENCES

(1) Sharma L, Kapoor D, Issa S. Epidemiology of osteoarthritis: an update. Curr Opin Rheumatol 2006 Mar;18(2):147-56.

(2) Hunter DJ, Felson DT. Osteoarthritis. BMJ 2006 Mar 18;332(7542):639-42.

(3) Fransen M, McConnell S, Bell M. Exercise for osteoarthritis of the hip or knee. Cochrane Database Syst Rev 2003;(3):CD004286.

(4) Zhang W, Nuki G, Moskowitz RW, Abramson S, Altman RD, Arden NK, et al. OARSI recommendations for the management of hip and knee osteoarthritis: part III: Changes in evidence following systematic cumulative update of research published through January 2009. Osteoarthritis Cartilage 2010 Apr;18(4):476-99.

(5) Jordan KM, Arden NK, Doherty M, Bannwarth B, Bijlsma JW, Dieppe P, et al. EULAR Recommendations 2003: an evidence based approach to the management of knee osteoarthritis: Report of a Task Force of the Standing Committee for International Clinical Studies Including Therapeutic Trials (ESCISIT). Ann Rheum Dis 2003 Dec;62(12):1145-55.

(6) Petersen AM, Pedersen BK. The anti-inflammatory effect of exercise. J Appl Physiol 2005 Apr;98(4):1154-62.

(7) Helmark IC, Mikkelsen UR, Borglum J, Rothe A, Petersen MC, Andersen O, et al. Exercise increases interleukin-10 levels both intraarticularly and peri-synovially in patients with knee osteoarthritis: a randomized controlled trial. Arthritis Res Ther 2010 Jul 1;12(4):R126.

(8) HOLLANDER JL, BROWN EM, Jr., JESSAR RA, BROWN CY. Hydrocortisone and cortisone injected into arthritic joints; comparative effects of and use of hydrocortisone as a local antiarthritic agent. J Am Med Assoc 1951 Dec 22;147(17):1629-35.

(9) Ostergaard M, Halberg P. Intra-articular corticosteroids in arthritic disease: a guide to treatment. BioDrugs 1998 Feb;9(2):95-103.

(10) Arroll B, Goodyear-Smith F. Corticosteroid injections for osteoarthritis of the knee: meta-analysis. BMJ 2004 Apr 10;328(7444):869.

(11) Ostergaard M, Halberg P. [Intra-articular glucocorticoid injections in joint diseases]. Ugeskr Laeger 1999 Feb 1;161(5):582-6.

(12) van Baar ME, Dekker J, Oostendorp RA, Bijl D, Voorn TB, Bijlsma JW. Effectiveness of exercise in patients with osteoarthritis of hip or knee: nine months' follow up. Ann Rheum Dis 2001 Dec;60(12):1123-30.

(13) Roos EM, Toksvig-Larsen S. Knee injury and Osteoarthritis Outcome Score (KOOS) - validation and comparison to the WOMAC in total knee replacement. Health Qual Life Outcomes 2003;1(May 25: 17).
